# Generation and analysis of recombinant Bunyamwera orthobunyaviruses expressing V5 epitope-tagged L proteins

**DOI:** 10.1099/vir.0.007567-0

**Published:** 2009-02

**Authors:** Xiaohong Shi, Richard M. Elliott

**Affiliations:** Centre for Biomolecular Sciences, School of Biology, University of St Andrews, North Haugh, St Andrews, Scotland KY16 9ST, UK

## Abstract

The L protein of Bunyamwera virus (BUNV; family *Bunyaviridae*) is an RNA-dependent RNA polymerase, 2238 aa in length, that catalyses transcription and replication of the negative-sense, tripartite RNA genome. To learn more about the molecular interactions of the L protein and to monitor its intracellular distribution we inserted a 14 aa V5 epitope derived from parainfluenza virus type 5, against which high-affinity antibodies are available, into different regions of the protein. Insertion of the epitope at positions 1935 or 2046 resulted in recombinant L proteins that retained functionality in a minireplicon assay. Two viable recombinant viruses, rBUNL4V5 and rBUNL5V5, expressing the tagged L protein were rescued by reverse genetics, and characterized with respect to their plaque size, growth kinetics and protein synthesis profile. The recombinant viruses behaved similarly to wild-type (wt) BUNV in BHK-21 cells, but formed smaller plaques and grew to lower titres in Vero E6 cells compared with wt BUNV. Immunofluorescent staining of infected cells showed the L protein to have a punctate to reticular distribution in the cytoplasm, and cell fractionation studies indicated that the L protein was present in both soluble and microsomal fractions. Co-immunoprecipitation and confocal microscopic assays confirmed an interaction between BUNV L and N proteins. The recombinant viruses expressing tagged L protein will be highly valuable reagents for the detailed dissection of the role of the BUNV L protein in virus replication.

## INTRODUCTION

The family *Bunyaviridae* contains more than 300 members and is divided into five genera, *Orthobunyavirus*, *Hantavirus*, *Nairovirus*, *Phlebovirus* and *Tospovirus*. Several members are important human pathogens, such as La Crosse orthobunyavirus (LACV), Hantaan hantavirus, Rift Valley fever phlebovirus and Crimean-Congo hemorrhagic fever nairovirus. Bunyamwera virus (BUNV) is the prototype of both the genus *Orthobunyavirus* and the family. Like other members of the family *Bunyaviridae*, BUNV possesses a tripartite, negative-sense RNA genome that encodes four structural proteins. The largest segment (L) codes for the L protein, an RNA-dependent RNA polymerase (RdRp); the medium segment (M) codes for a precursor that is co-translationally cleaved into the two glycoproteins (Gn and Gc) and a non-structural protein (NSm); and the small segment (S) codes for the nucleoprotein (N) and a second non-structural protein (NSs) in overlapping reading frames (Elliott, 1996; Nichol *et al.*, 2005; Schmaljohn & Hooper, 2001). Bunyaviruses replicate in the cytoplasm of infected cells, and assemble and bud in the Golgi complex (Kuismanen *et al.*, 1982; Murphy *et al.*, 1973; Schmaljohn & Hooper, 2001).

The bunyavirus L protein is the catalytic enzyme for replication of the RNA genome and transcription of viral mRNAs; only the L and N proteins are required for these processes (Dunn *et al.*, 1995; Lopez *et al.*, 1995). In common with all negative-strand RNA viruses, the viral genomic (−) and antigenomic (+) RNAs are complexed with the N protein to form ribonucleoproteins (RNP) that serve as templates for viral transcription and replication (Ortin & Parra, 2006). The BUNV L protein is 259 kDa and contains 2238 aa (Elliott, 1989). Although no extended homology is found with other negative-stranded virus polymerases or even with the polymerases of viruses in other genera in the family *Bunyaviridae*, the motifs conserved in other RdRps that comprise the ‘polymerase module’ have been identified (Aquino *et al.*, 2003; Elliott, 1989; Gowda *et al.*, 1998; Muller *et al.*, 1994; Poch *et al.*, 1989; Quinan *et al.*, 2008; Roberts *et al.*, 1995; van Poelwijk *et al.*, 1997). Its role as the viral polymerase was confirmed by the ability of the L protein to transcribe authentic BUNV RNP templates (Jin & Elliott, 1991).

Few studies have reported the intracellular location of bunyavirus polymerase proteins. LACV RNA synthesis was demonstrated to occur in the cytoplasm by RNA pulse-labelling and cell fractionation (Rossier *et al.*, 1986), implying the presence of the L protein. The L protein of Toscana phlebovirus (TOSV) was observed to have a punctate staining pattern in the cytoplasm of infected cells as detected with a monospecific antibody raised against a bacterially expressed fragment of the L protein (Di Bonito *et al.*, 1999; Kukkonen *et al.*, 2004), while transient expression of the Tula hantavirus (TULV) L protein fused to green fluorescent protein (GFP) also showed a cytoplasmic perinuclear distribution (Di Bonito *et al.*, 1999; Kukkonen *et al.*, 2004). The ‘viral factory’ assembled by BUNV was described and characterized (Novoa *et al.*, 2005; Salanueva *et al.*, 2003); the factory contains novel tubular structures with a globular head, and electron microscopical studies showed the presence of the BUNV L protein and dsRNA in the globular head (Fontana *et al.*, 2008).

To elucidate further the biological function of the bunyavirus RNA polymerase, the BUNV L protein was tagged with a 14 aa V5 epitope derived from the parainfluenza virus type 5 protein (Southern *et al.*, 1991) and two recombinant viruses (rBUNV-L4V5 and rBUNV-L5V5) expressing tagged L protein were rescued by reverse genetics. We demonstrated that the tagged L protein interacted with the viral N protein and formed punctate to reticular membranous structures in the cytoplasm of virus-infected cells. The effect of the V5 insertion on replication was also assessed with the two recombinant viruses.

## METHODS

### Cells and viruses.

Vero E6 (ATCC C1008), BHK-21 and BSR-T7/5 (Buchholz *et al.*, 1999) cells were maintained as described previously (Shi *et al.*, 2005). Working stocks of wild-type (wt) and recombinant BUNV were grown in BHK-21 cells and titres were determined by plaque assay as detailed previously (Watret *et al.*, 1985).

### Antibodies.

Anti-N, a rabbit antiserum against BUNV nucleocapsid protein, was described previously (Lappin *et al.*, 1994; Weber *et al.*, 2001). An anti-V5 mAb was purchased from Serotec. A rabbit polyclonal antibody against GM130, a *cis*-Golgi matrix protein (Nakamura *et al.*, 1995), was provided by Dr M. Lowe (School of Biological Sciences, University of Manchester, UK). Goat anti-rabbit antibody conjugated to fluorescein isothiocyanate was purchased from Sigma, and goat anti-mouse antibody conjugated to Cy5 was purchased from Amersham Phamacia Biotech.

### Plasmids.

Plasmids that either express BUNV proteins (pTM1-BUNL for expressing L protein, and pTM1-BUNS for expressing N and NSs), or generate full-length antigenomic RNA transcripts [pT7riboBUNL(+), pT7riboBUNM(+), pT7riboBUNS(+)] have been described previously (Bridgen & Elliott, 1996; Lowen *et al.*, 2004), as have the BUNV-derived minigenome expressing construct, pT7riboBUNMREN(−), that contains the *Renilla* luciferase gene in the negative-sense (Weber *et al.*, 2001), and the control plasmid pTM1-FF-Luc that expresses firefly luciferase (Weber *et al.*, 2002).

**Fig. 1. f1:**
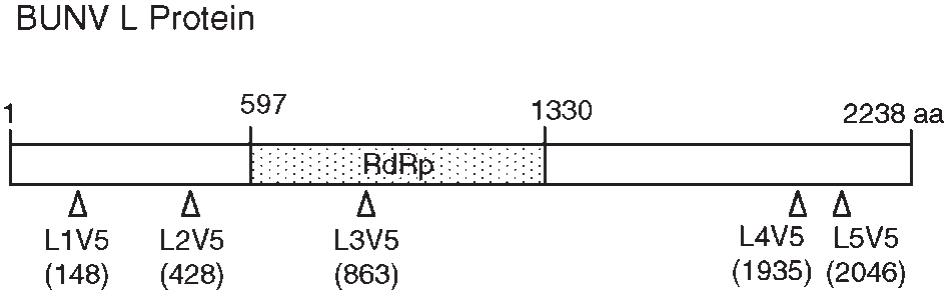
Construction of the V5 epitope-tagged BUNV L protein. The 14 aa V5 epitope was inserted into the coding region of the L proteins by using PCR mutagenesis with both pTM1-BUNL and pT7riboBUNL DNA as template. The insertion sites are indicated by triangles with the residue number shown below and were designated L1V5–L5V5. The dotted area represents the predicated catalytic RdRp domains of the BUNV L protein.

The V5 epitope (GKPIPNPLLGLDST) (Southern *et al.*, 1991) was inserted into the BUNV L protein coding region in both pTM1-BUNL and pT7riboBUNL(+) as shown in Fig. 1 by using a PCR mutagenesis approach (Shi & Elliott, 2002). Ten V5-tagged BUNV L mutants were constructed, five derived from pTM1-BUNL (designated pTM1-BUNL1V5–pTM1-BUNL5V5) and five based on pT7riboBUNL(+) (pT7riboBUNL1V5–pT7riboBUNL5V5). All constructs were confirmed by DNA sequence analysis. The primers used and details of PCR amplification are available upon request.

### Indirect immunofluorescence staining.

Immunofluorescence assays were performed as described previously (Shi & Elliott, 2002). Briefly, transfected or infected BSR-T7/5 cells grown on glass coverslips of 13 mm diameter were fixed with 4 % paraformaldehyde in PBS and permeabilized with 0.1 % Triton X-100 in PBS before staining with the specific primary antibodies and secondary antibody conjugates. Localization of fluorescently labelled proteins was examined using either a Zeiss LSM confocal microscope or DeltaVision 3.5 Restoration Microscope (Applied Precision) as indicated in the Figure legends. The DeltaVision images were processed with deconvolution software (softWoRx; Applied Precision) to remove the non-specific noise in the images.

### BUNV minireplicon assay.

The BUNV minireplicon assay was performed as described previously (Kohl *et al.*, 2004) with minor modifications. Briefly, BSR-T7/5 cells were transfected with pTM1-BUNS (0.1 μg) and pTM1-BUNL (0.3 μg), or one of the mutated L segment cDNAs cloned into pTM1 (also 0.3 μg), together with the minigenome plasmid, pT7riboBUNMREN(−) (0.3 μg), and pTM1-FF-Luc (0.1 μg) (internal transfection control). At 24 h post-transfection, *Renilla* and firefly luciferase activities were measured using the Dual-Luciferase Assay kit (Promega) according to the manufacturer's instruction.

### Subcellular fractionation by ultracentrifugation.

Preparation of total microsomal fractions of rBUNL4V5-infected BHK-21 cells was performed as described previously (Ehrenreich *et al.*, 1973) with modifications. Briefly, BHK-21 cells grown in a 175 cm^2^ flask were infected at an m.o.i. of 1.0 p.f.u. per cell and incubated for 36 h. Cells were washed twice with cold PBS and once with 0.25 mM sucrose/10 mM HEPES buffer (pH 7.2) by centrifugation at 700 ***g*** for 3 min at 4 °C. The cell pellet was resuspended in 1.0 ml 0.25 mM sucrose/10 mM HEPES buffer and disrupted by three freeze–thaw cycles on dry ice–37 °C, followed by three 10 s pulses of sonication in a water bath at 4 °C, and then 10 strokes in a glass homogenizer. After clarification at 1000 ***g*** for 10 min at 4 °C, the supernatant (total fraction, T) was further centrifuged at 65 000 r.p.m. for 15 min (Beckman TL-100 rotor) to produce a soluble fraction (S) and pellet (microsomal fraction, Mi). Samples from all fractions were analysed by SDS-PAGE and Western blotting.

### Metabolic radiolabelling and immunoprecipitation.

Metabolic radiolabelling and immunoprecipitation of BUNV proteins were performed as described previously (Shi *et al.*, 2005). Briefly, cells were labelled with [^35^S]methionine (Amersham Pharmacia Biotech) at various time points post-infection or at 16 h post-transfection as indicated in the Figure legends. The labelled cells were either analysed directly by SDS-PAGE or lysed for immunoprecipition on ice with 300 μl non-denaturing RIPA buffer (50 mM Tris/HCl, pH 7.4, 1 % Triton X-100, 300 mM NaCl, 5 mM EDTA) containing a cocktail of protease inhibitors (Roche). BUNV proteins were immunoprecipitated with anti-N or anti-V5 antibodies that had been conjugated to Protein A–agarose (Sigma). The beads were washed with RIPA buffer containing 0.1 % Triton X-100 and once with ice-cold PBS, and the bound proteins were analysed by SDS-PAGE under reducing conditions.

### Virus rescue by reverse genetics.

Rescue experiments were performed as described previously (Lowen *et al.*, 2004). Briefly, BSR-T7/5 cells were transfected with a mixture of three plasmids: 1.0 μg each pT7riboBUNM(+), pT7riboBUNS(+) and either pT7riboBUNL(+) or one of the pT7riboBUNL(+)-derived V5-tagged L cDNA mutants. At 6 h post-transfection, 4 ml growth medium was added and incubation continued. Rescues were attempted at least twice for each construct, and transfected cells were maintained for up to 14 days or until cytopathic effects (CPE) were evident. The transfectant viruses were isolated by plaque formation on Vero E6 cells.

## RESULTS

### Generation of V5-tagged BUNV L protein

Although the BUNV L protein plays a key role in viral replication and transcription, it is the least characterized of all the structural proteins. Previously, we described the effects of some specific mutations on the activity of the BUNV L protein (Dunn *et al.*, 1995; Jin & Elliott, 1992), and produced monospecific antibodies that recognized the L protein in immunoprecipitation and Western blotting procedures (Jin & Elliott, 1992). Unfortunately, although these antibodies recognize the L protein by immunogold labelling of thin sections of glutaraldehyde-fixed infected cells and electron microscopy (Fontana *et al.*, 2008), they do not detect the L protein when used for immunofluorescent staining of infected cells (unpublished observations), thus limiting investigations on the intracellular distribution of the L protein. Therefore, we attempted to modify the L protein by the addition of specific epitopes; however, fusing the Flag-tag to either the N or C terminus of the L protein resulted in severe loss of activity in our minireplicon assay (F. Weber & R. M. Elliott, unpublished data). Prompted by the report of Duprex *et al.* (2002) that the measles virus L protein could tolerate insertion of GFP and retain function, we targeted internal regions of BUNV L for the insertion of the V5 epitope from parainfluenza virus type 5 (Southern *et al.*, 1991). Based on alignment of the L protein amino acid sequences from three orthobunyaviruses (BUNV, LACV and oropouche virus) and bioinformatic predictions of the BUNV L protein using programs in the protein structure server scratch (http://www.ics.uci.edu/∼baldig/scratch/) (Cheng *et al.*, 2005), the following criteria were considered to determine sites for possible insertion: regions of higher variability, regions predicted to have fewer residue contacts and relative higher solvent accessibility, and regions that avoided predicted *α*-helix and *β*-strand structures (see Supplementary Fig. S1 available in JGV Online). Five sites were identified that fulfilled these criteria and plasmids expressing the V5-tagged BUNV L proteins were constructed (Fig. 1).

### Intracellular localization and polymerase activity of V5-tagged L proteins

**Fig. 2. f2:**
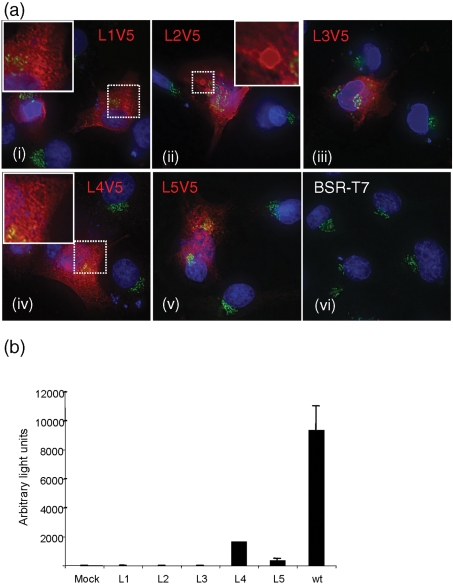
Characterization of the V5-tagged BUNV L protein. (a) Intracellular localization of the V5-tagged L proteins in BSR-T7/5 cells. Cells were transfected with 0.5 μg each pTM1-based construct as indicated [panels (i) to (v)] or not transfected [panel (vi)]. Cells were fixed with 4 % paraformaldehyde, co-stained with anti-V5 mAb and anti-GM130 antibodies and examined using the DeltaVision restoration microscope (Applied Precision). Only the merged images are shown. The insets in panels (i), (ii) and (iv) are enlarged from the selected regions. V5-tagged L proteins stained red, the Golgi stained green and cell nuclei stained with 4’6’-diamidino-2-phenylindole (DAPI) are shown in blue. (b) Activity of V5-tagged L proteins in the minireplicon assay. BSR-T7/5 cells were transfected with pTM1-BUNS, pT7riboBUNMRen(−) and either wt pTM1-BUNL or V5-tagged L mutants. *Renilla* luciferase activity in cell lysates was measured at 24 h post-transfection and is shown in arbitrary light units. Mock, negative control without L cDNA; L1, pTM1-L1V5; L2, pTM1-L2V5; L3, pTM1-L3V5; L4, pTM1-L4V5; L5, pTM1-L5V5; and wt, pTM1-BUNL.

The accessibility of the V5 epitope in the recombinant L proteins was assessed by immunofluorescent staining of plasmid-transfected cells (Fig. 2a[Fig f2]). Except for L2V5, all V5-tagged L proteins displayed a cytoplasmic, punctate to reticular staining pattern [Fig. 2a[Fig f2], panels (i), (iii)–(v)] [clearly shown in the magnified insets in panels (i) and (iv)], suggesting that the L protein may be associated with intracellular membrane compartments. When the V5 epitope was inserted at position 428 (construct L2V5), a different staining pattern was observed that was suggestive of the aggregation of misfolded protein.

The effect of these insertions on the functionality of L protein was assessed using the minireplicon reporter assay on BSR-T7/5 cells. As shown in Fig. 2(b), insertions of the V5 tag at positions 148 (L1V5), 428 (L2V5) and 863 (L3V5) abolished polymerase activity in the minireplicon assay. Polymerases with insertions towards the C terminus at positions 1935 (L4V5) and 2046 (L5V5) were still functional, though with reduced activity compared with that of wt BUNV L of 18 (L4V5) and 4 % (L5V5), respectively.

### Rescue of recombinant viruses expressing V5-tagged L protein

The five recombinant L cDNAs were next used for virus rescue to assess the effect of the V5 insertion on virus viability (Lowen *et al.*, 2004). Consistent with the results from the minireplicon assay, recombinant viruses were recovered from the two functional V5-tagged L constructs, pT7riboBUNL(+)-L4V5 and -L5V5 (the rescued viruses were designated rBUNL4V5 and rBUNL5V5, or rL4V5 and rL5V5 for short), whereas no virus was rescued from the non-functional constructs pT7riboBUNL(+)-L1V5, -L2V5 and -L3V5.

**Fig. 3. f3:**
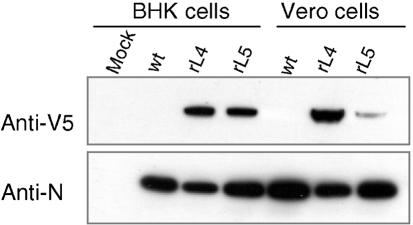
Characterization of recombinant viruses expressing epitope-tagged L protein. Western blot analysis of BHK-21 and Vero E6 cells infected with recombinant viruses rBUNL4V5 (rL4), rBUNL5V5 (rL5) or wt BUNV (wt) at an m.o.i. of 1 p.f.u. per cell. Extracts from equivalent numbers of cells at 36 h post-infection were loaded into each well of a 4–12 % NuPage gel (Invitrogen) and the blot was probed with anti-V5 and anti-N antibodies.

The presence of V5 tag in the L protein of the recombinant viruses was confirmed by Western blotting analysis of infected BHK-21 and Vero E6 cells (Fig. 3). The L proteins of rBUNL4V5 and rBUNL5V5 were detected by anti-V5 mAb, whereas no signal was seen in lysates of cells infected with wt BUNV control. The N protein was also clearly detected in cells infected by both wt BUNV and the V5-tagged mutant viruses (Fig. 3).

### Effect of V5 insertions of the L protein on rBUNV growth and protein synthesis in cell culture

**Fig. 4. f4:**
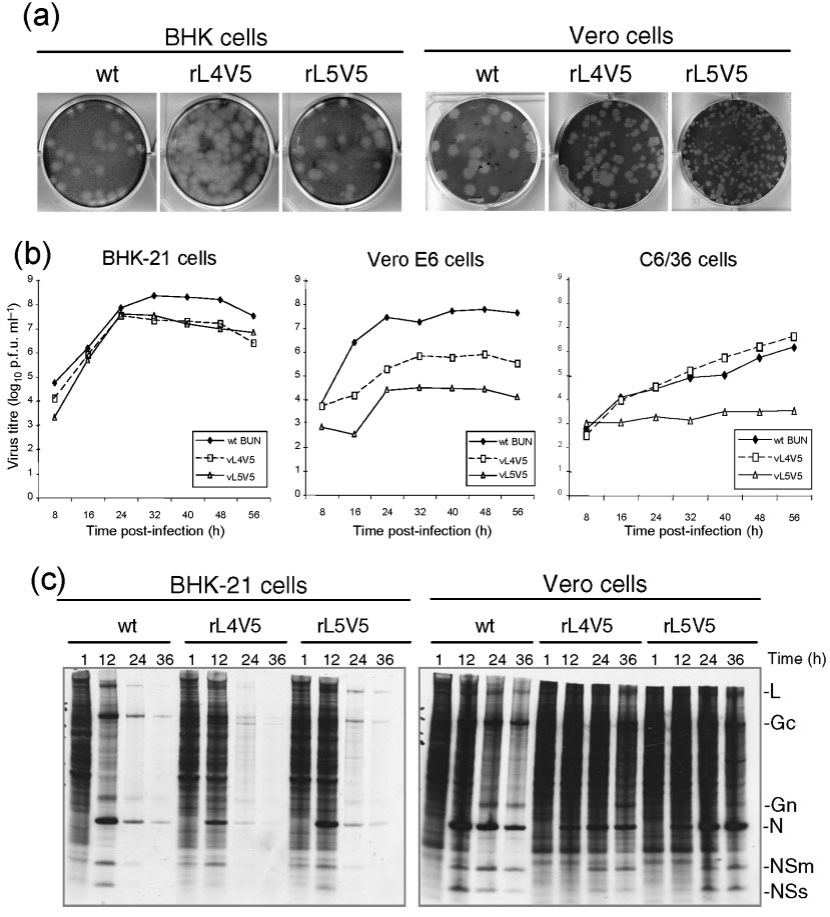
Plaque phenotype, growth kinetics and protein synthesis profile of wt and recombinant viruses. (a) Comparison of plaque size on BHK-21 and Vero E6 cells. Cell monolayers were fixed with 4 % formaldehyde and stained with Giemsa solution. (b) Viral growth curves on BHK-21, Vero E6 and *Aedes albopictus* C6/36 cells. Cells were infected with either wt or recombinant viruses at an m.o.i. of 0.01 p.f.u. per cell. Virus was harvested at 8 h intervals and titrated by plaque assay. (c) Time-course of protein synthesis on BHK-21 and Vero E6 cells. Cells infected at an m.o.i. of 1.0 p.f.u. per cell were labelled with 80 μCi (2.96 MBq) [^35^S]methionine for 20 min at the time points indicated and cell lysates analysed by 15 % SDS-PAGE. The positions of the viral proteins are indicated at the right.

The effects of the V5 insertion in the viral polymerase on virus viability, plaque size, growth kinetics and the ability to shut-off host protein synthesis were compared with those of wt BUNV. As shown in Fig. 4, insertion of the V5 epitope had differential impacts on virus growth in BHK-21 and Vero E6 cells. In BHK-21 cells, the plaques formed by rL4V5, rL5V5 and wt BUNV were similar in size (Fig. 4a[Fig f4]). This was consistent with the virus growth kinetics on BHK-21 cells, in which the titre of two recombinant viruses released into the medium were comparable to that of wt BUNV up to 24 h post-infection, though declined slightly thereafter (Fig. 4b[Fig f4]). Metabolic labelling of infected BHK-21 cells with [^35^S]methionine showed that the degree of shut-off of host cell protein synthesis by the two recombinant viruses was slightly slower than wt BUNV at 12 h post-infection, but was complete by 24 h (Fig. 4c[Fig f4], left panel).

On Vero E6 cells, however, the recombinant viruses formed smaller plaques than those of wt BUNV, with rL5V5 plaques being considerably smaller (Fig. 4a[Fig f4]). The recombinants grew much slower and to lower titres than wt BUNV (Fig. 4b[Fig f4], central panel), in particular rL5V5 displayed the poorest growth with virus titres nearly 1000-fold less, while rL4V5 gave titres about 100-fold less, across the whole infection period. In addition, shut-off of host protein synthesis by rL4V5 and rL5V5 was obviously delayed compared with wt BUNV (Fig. 4c[Fig f4]).

We also investigated the growth of two recombinant viruses on *Aedes albopictus* C6/36 (mosquito) cells. As BUNV does not cause CPE or form plaques, and does not cause marked host cell protein shut-off in insect cells (Elliott & Wilkie, 1986), only virus growth kinetics were examined. Notably, although rL4V5 grew nearly as efficiently as wt BUNV, with virus titre reaching over 10^6^ p.f.u. ml^−1^, the growth of rL5V5 was severely retarded, with titres of released virus only about 10^3^ p.f.u. ml^−1^ throughout the infection period (Fig. 4b[Fig f4]). The results suggest that mutations in the viral polymerase gene affect the ability of BUNV to replicate in different cells.

### Membrane association of the BUNV L protein

**Fig. 5. f5:**
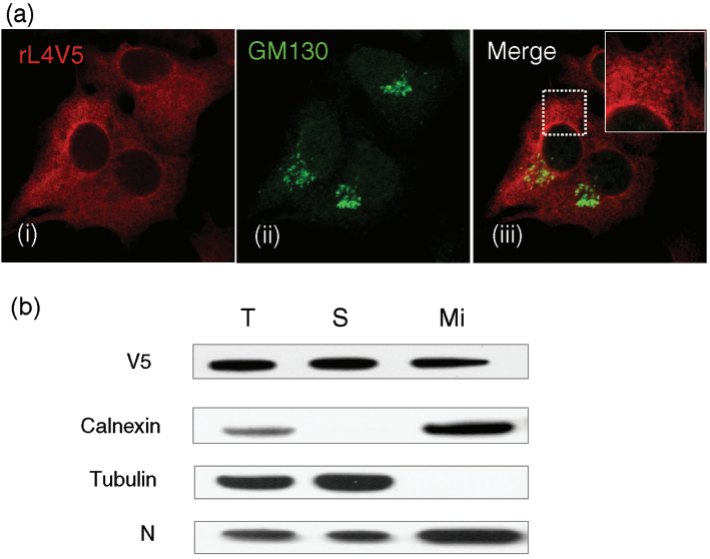
Membrane association of viral proteins. (a) Punctate staining pattern of the BUNV L protein in rBUNL4V5-infected BSR-T7/5 cells. Cells were infected with rBUNL4V5 at an m.o.i. of 5, fixed and then co-stained with anti-V5 (red) and anti-GM130 (Golgi marker, green) antibodies. An enlarged area from panel (iii) is also shown. (b) Western blotting analysis of subcellular fractions of rBUNL4V5-infected BHK-21 cells. Cells were infected with rBUNL4V5 at an m.o.i. of 5 p.f.u. per cell. Cells were homogenized and fractionated as described in Methods. The presence of the L and N proteins was determined by Western blotting with anti-V5 and anti-BUNV N antibodies as appropriate. Tubulin and calnexin were used as soluble and membrane protein markers, respectively, and detected with appropriate antibodies. T, Total cell lysate; S, soluble fraction and Mi, microsomal fractions.

The distribution of the L protein in rBUNL4V5-infected cells resembled that seen in plasmid-transfected cells (Fig. 2a[Fig f2]), with the L protein located cytoplasmically, often concentrated in the perinuclear region, and exhibiting a punctate to reticular staining pattern [Fig. 5a[Fig f5], panel (i)]. Although bunyaviruses mature in the Golgi apparatus, no obvious co-localization was observed between the L protein and the Golgi marker GM130 in recombinant virus-infected cells [Fig. 5a[Fig f5], panels (ii) and (iii)]. Similar observations were made with rBUNL5V5-infected cells (data not shown).

The staining pattern exhibited by the V5-tagged L protein suggested that the protein was likely to be associated with intracellular membrane structures. To confirm the membrane binding property of the BUNV polymerase, homogenized samples of virus-infected cells were fractionated by ultracentrifugation into three fractions, total (T), soluble (S) and microsomal (Mi), and were subjected to Western blot analysis. This revealed that both BUNV L and N proteins were present in both soluble and microsomal fractions (Fig. 5b[Fig f5]). As control for the fractionation procedure, the endoplasmic reticulum integral membrane protein calnexin (membrane marker) and tubulin (soluble marker) were shown to be present only in either microsomal or soluble fractions, respectively. The presence of both N and L proteins in the membrane fraction suggests the involvement of membrane structures in BUNV replication.

### Interaction between BUNV L and N proteins

**Fig. 6. f6:**
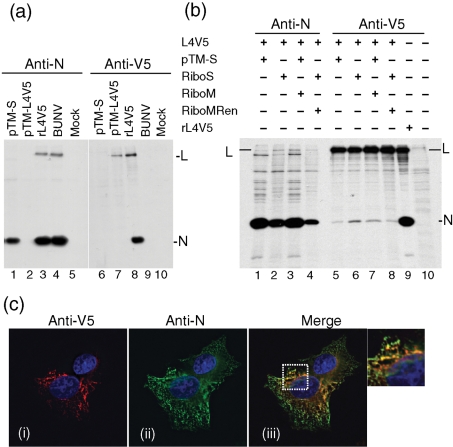
Interaction of the BUNV L and N proteins. (a and b) Co-immunoprecipitation assays. BSR-T7/5 cells were either transfected with cDNA constructs or infected with wt BUNV or recombinant viruses as indicated. Cells were labelled with [^35^S]methionine and were immunoprecipitated with either anti-N or anti-V5 antibodies. The positions of the BUNV L and N proteins are indicated at the right. (c) DeltaVision microscopic images of BSR-T7/5 cells infected with rBUNL4V5. Cells were stained with anti-V5 [red, panel (i)] and anti-N [green, panel (ii)] antibodies, and merged images are shown in panel (iii). Co-localization is shown in yellow. Nuclei were stained blue with DAPI. An enlarged area from panel (iii) is also shown.

We previously observed that the BUNV L protein was co-immunoprecipitated with the N protein in lysates of BUNV-infected cells (Shi *et al.*, 2006), and this was confirmed as shown in Fig. 6: the L protein in both rBUNL4V5- and wt BUNV-infected cells was precipitated by anti-N antiserum (Fig. 6a[Fig f6], lanes 3 and 4). The interaction between the L and N proteins was also demonstrated by co-precipitation with anti-V5 antibody in which a substantial amount of the N protein was co-precipitated from rBUNL4V5-infected cells; however, as expected, no proteins were precipitated from wt BUNV-infected cells (Fig. 6a[Fig f6], lanes 8 and 9). As controls, only the N protein or the V5-tagged L protein was immunoprecipitated from cells transfected with the corresponding individual cDNA constructs (Fig. 6a[Fig f6], lanes 1 and 7).

The interaction of the L and N proteins was further investigated in BSR-T7/5 cells co-transfected with L and N protein-expressing cDNAs (Fig. 6b[Fig f6]). However, in this case the protein recognized by its cognate antibody was precipitated more efficiently than the other co-expressed protein (e.g. compare lanes 1 and 5 with lane 9). Since this difference may indicate that the interaction of the L and N proteins (as in the case of transiently expressed proteins) was relatively weak compared with N and L interaction in RNP complexes (as would occur in virus-infected cells), we next assessed whether co-expression of a viral transcript in the transfected cells would increase the amount of co-precipitating protein. Therefore, cells were transfected with pT7riboBUNS as a source of both N protein and full-length S segment antigenome RNA (as used in the rescue protocol; Lowen *et al.*, 2004) or additionally transfected with either pT7riboBUNM(+), which expresses full-length antigenome M segment RNA, or the minigenome plasmid pT7riboBUNMREN(−), which expresses a negative-sense M segment-like RNA containing the *Renilla* luciferase gene, thus reconstituting a minireplicon assay (Weber *et al.*, 2002). The amounts of protein in each band were estimated by scanning the gel in a phosphorimager (FLA-5000; Fujifilm Corporation) and use of density analysis software (Image Gauge, version 4.21; Fujifilm Corporation and Koshin Graphic Systems, Inc.), and by normalizing the amount of protein expressed in the different samples, the relative degree of co-precipitation could be assessed (see Supplementary Table S1 available in JGV Online). In cells transfected with pT7riboBUNS, which expresses about fourfold less N protein than pTM1-BUNS (compare lanes 1 and 2), about eightfold more L protein was co-precipitated with anti-N antibody, and about fivefold more N protein was co-precipitated by the anti-V5 antibody (compare lanes 5 and 6). The co-transfection of pT7riboBUNM(+) (lanes 3 and 7) or pT7riboBUNMREN(−) (lanes 4 and 8) also increased the co-precipitation of the L and N proteins. This suggests that the assembly of RNPs on viral RNA templates magnifies the observed interaction between L and N compared with the situation where no RNPs are present in the cell.

The association between the L and N proteins was also visualized in rL4V5-infected BSR-T7/5 cells viewed using the DeltaVision microscope followed by processing of the images with deconvolution software (Fig. 5c[Fig f5]). Both proteins were observed throughout the cytoplasm with L being more perinuclearly localized, whereas N showed a more general cytoplasmic distribution. The majority of the L protein appeared to co-localize with N, forming a network structure [see the enlarged region in Fig. 6c[Fig f6] panel (iii) with co-localizaion shown in yellow]. These images are again consistent with L and N interaction occurring on intracellular membranes.

## DISCUSSION

The bunyavirus L protein, like the polymerases of other negative-strand viruses, plays a key role in the virus life cycle, including genome replication and mRNA transcription. Study of some aspects of the BUNV L protein has been impeded by the lack of an antibody that can recognize the native or functional protein in cells by immunofluorescent detection methods, as the antibodies previously raised against BUNV L protein were only able to detect the protein by immunoprecipitation and Western blotting (Jin & Elliott, 1992), or by immunogold labelling of thin sections of fixed infected cells and electron microscopy (Fontana *et al.*, 2008). Therefore, we attempted to introduce an epitope tag into the L protein and determined whether the recombinant polymerase proteins would be (i) functional and (ii) recognized by the cognate antibody. To this end, we inserted the V5 epitope at different positions in the coding region of the BUNV L protein based on sequence comparison and bioinformatic predictions (Fig. 1). Insertion at two positions towards the C terminus of L resulted in recombinant proteins that retained functionality in a minireplicon assay, and in turn could be incorporated into infectious viruses.

Little is known about the domain structure of bunyavirus polymerases. Muller *et al.* (1994) compared the L protein sequences of bunyaviruses and arenaviruses (the other family of cytoplasmic-replicating, segmented genome, negative-sense RNA viruses) and identified three conserved regions, two near the N terminus (regions 1 and 2) and one in the centre (region 3). The C termini of the proteins were found to be more variable. Insertion of the V5 epitope at position 148 (construct L1V5) in region 1 of the BUNV L protein appeared not to disrupt the structure of the protein, as the intracellular distribution of L1V5 was indistinguishable from that of the two functional constructs L4V5 and L5V5. However, the abolition of polymerase activity by V5 insertion at this position indicated the importance of this region in virus replication, though a specific function has yet to be ascribed to region 1.

Construct L2V5 has the epitope inserted at position 428, situated between regions 1 and 2, and there is no significant conservation of amino acids among the L proteins of viruses in the five bunyavirus genera in this area. However, perturbation of this ‘linking’ region resulted in misfolding of the BUNV L protein, suggesting that the region may be required for maintaining polymerase structure. Insertion of V5 at position 863 (construct L3V5), which is in a variable domain upstream of region 3 (which contains the polymerase module present in all the RdRps), also abolished polymerase activity. However, V5 insertion at positions 1935 and 2046 (constructs L4V5 and L5V5) had no lethal impact on polymerase activity, and two recombinant viruses expressing tagged L protein were recovered, suggesting that the variable C-terminal region of BUNV polymerase is more tolerant of mutation than other regions.

Comparison of the recombinant viruses expressing tagged L protein and wt BUNV revealed that the viruses behaved differentially in different cell lines with regards to plaque size, growth kinetics, and protein synthesis profile. Cell-type-dependent effects on virus growth suggest that the V5 insertion in the L protein likely affects the interaction of the viral polymerase with host factors required for replication. Cell-type dependence for mRNA synthesis was reported for LACV (Raju *et al.*, 1989), and it was described recently that a host factor that bound to the polymerase of tomato spotted wilt virus, a plant-and-insect-infecting bunyavirus, was able to render human cell lines permissive for virus replication (Medeiros *et al.*, 2005). Our data suggest that the C-terminal region of the BUNV L protein, especially the region around residue 2046 (the site of insertion of the epitope in construct L5V5) is perhaps involved in the interaction with a cellular co-factor(s). Experiments are ongoing to identify cellular proteins that interact with the epitope-tagged L proteins.

Bunyaviruses replicate in the cytoplasm of infected cells (Kuismanen *et al.*, 1982; Murphy *et al.*, 1973; Schmaljohn & Hooper, 2001). In this study, the L protein was clearly shown distributed in the cytoplasm with a punctate to reticular staining pattern in cells that were either transfected with functional V5-tagged L segment cDNAs or infected by recombinant viruses; in some cells there was a concentration of staining in the perinuclear region. A cytoplasmic punctate staining pattern was also reported for the L protein of TOSV, though the TOSV L antibodies also recognized smaller forms of L, perhaps derived from defective RNAs. Hence the localization of full-length, functional L protein was unclear (Di Bonito *et al.*, 1999). For TULV, the L protein antibodies also failed to recognize the protein in immunofluorescent assays (Kukkonen *et al.*, 2004), but a TULV L–GFP fusion protein was observed in the perinuclear region of plasmid-transfected cells, though the functional significance of this could not be ascertained (Kukkonen *et al.*, 2004, 2005). Thus, our data with epitope-tagged recombinant BUNV represent the bona fide picture of viral polymerase distribution during virus infection.

The punctate expression pattern of TULV L–GFP was interpreted as indicating an association of the protein with intracellular membranes, and indeed the TULV L protein was found in the microsomal membrane fraction of infected cells (Kukkonen *et al.*, 2004). The V5-tagged BUNV L, as well as the N protein, was detected in both cytosolic and microsomal fractions (Fig. 5). We suggest that the cytosolic fraction contains newly made soluble proteins, while the membrane-associated proteins represent replication complexes. This is consistent with the observations of Fontana *et al.* (2008) that also suggest the BUNV replication complex is associated with intracellular membrane compartments. Further characterization of these membranes is ongoing, and the epitope-tagged L protein expressing viruses will be valuable reagents in these studies. Furthermore, the success of tagging the BUNV L protein with the V5 epitope suggests it may be possible to insert longer sequences into the polymerase, such as GFP or other fluorescent tags, as has been achieved for the viral polymerases of measles, rinderpest and canine distemper viruses (Brown *et al.*, 2005; Duprex *et al.*, 2002; Silin *et al.*, 2007).

## Supplementary Material

[Supplementary Material]
