# Supplementary material for: Generation and analysis of recombinant Bunyamwera orthobunyaviruses expressing V5 epitope-tagged L proteins
Source: J Gen Virol. 2009 Feb;90(Pt 2):297–306. doi: 10.1099/vir.0.007567-0 (PMC2885054; doi:10.1099/vir.0.007567-0)
Supplement: [Supplementary Material] [file supp_90_2_297__index.html]

 Generation and analysis of recombinant Bunyamwera orthobunyaviruses expressing V5 epitope-tagged L proteins -- Shi and Elliott 90 (2): 297 Data Supplement - Supplementary Material -- Journal of General Virology

## 

### Generation and analysis of recombinant Bunyamwera orthobunyaviruses expressing V5 epitope-tagged L proteins, by X. Shi and R. M. Elliott

*Journal of General Virology* vol. **90**, part 2, pp. 297 - 306

**Supplementary Fig. S1.** (a) Alignment of the L proteins of bunyamwera, oropouche and La Crosse orthobunyaviruses. (b) Bioinformatic analysis of Bunyamwera virus L protein

**Supplementary Table S1.** Densitometric analysis of the BUNV N and L protein bands in Fig. 6(b)   
  
[Single PDF file]  (56 KB)

  
  
