# Supplementary material for: Generation and analysis of recombinant Bunyamwera orthobunyaviruses expressing V5 epitope-tagged L proteins
Source: J Gen Virol. 2009 Feb;90(Pt 2):297–306. doi: 10.1099/vir.0.007567-0 (PMC2885054; doi:10.1099/vir.0.007567-0)
Supplement: [Supplementary Material] [file supp_90_2_297__1.pdf]

(a)

```
ORO             MSQLLLNQYRNRILHCREPEIAKDIWRDLLNDRHNYFSREFCRAANLEYRNDVPAEDICA
BUN             MEDQAYDQYLHRIQAARTATVAKDISADILEARHDYFGRELCNSLGIEYKNNVLLDEIIL
LAC             MDYQEYQQFLARINTARDACVAKDIDVDLLMARHDYFGRELCKSLNIEYRNDVPFIDIIL
                *.     :*:   **   .*  . :****   *:*   **:**.**:*.:  .:**.*:*    :*

ORO             EVLDGYK--ARKVRFCTPDNYLLHDGKMYIIDFKVSVDDRSSRITREKYNEIFGEVFNPE
BUN             DVVPGVNLLNYNIPNVTPDNYIWDGHFLIILDYKVSVGNDSSEITYKKYTSLILPVMSEL
LAC             DIRPEVDPLTIDAPHITPDNYLYINNVLYIIDYKVSVSNESSVITYDKYYGLTRDISDRL
                ::     .     .      *****:    .   :  *:*:****.:  **  **  .**   :    :  .

                                      L1
ORO             GVDFEIVIIRLDPSNMTIHVDSRDFVNTIGPITLNISMQWFFDMKDFLFGKFRDDDKFHA
BUN             GIDTEIAIIRANPVTYQISIIGEEFKQRFPNIPIQLDFGRFFELRKMLLDKFADDEEFLM
LAC             SIPIEIVIVRIDPVSKDLHINSDRFKELYPTIVVDINFNQFFDLKQLLYEKFGDDEEFLL
                .:   **.*:* :*  .   :  :  .   *  :     *  ::::.:  .**::..:*    **  **::*

ORO             IISQGEFTMTLPWIEEDTPELLTHPIYNEFMSSMPEAEQALFKEALEFKSFGAEKWNIFL
BUN             MIAHGDFTLTAPWCTSDTPELEEHEIFQEFINSMPPRFVSLFKEAVNFSAYSSERWNTFL
LAC             KVAHGDFTLTAPWCKTGCPEFWKHPIYKEFKMSMPVPERRLFEESVKFNAYESERWNTNL
                 :::*:**:*  **     .  **:   *  *::**   ***       **:*:::*.::  :*.**   *

ORO             KGVMSKYGEYYKEFTKGHAHSIFLTTGDYPKPDKDQISAGWREMVNRVSSERDMSNDINQ
BUN             YRARAETEVDYNQFLSDKAHKIFMLEGDYMRPTQAEIDKGWELMSQRVYTEREIITDVTK
LAC             VKIREYTKKDYSEHISKSAKNIFLASGFYKQPNKNEISEGWTLMVGRVQDQREVSKSLHD
                          *.:.  .    *:.**:    *  *  .*  :  :*.  **    *    **    :*::  ..:  .

ORO             EKPSMHFIWAKN-DSNSNNNIQKLIKLSKSLQAMSGTGSYVNAFKSLGRLMDISSDVKKY
BUN             QKPSIHFIWVKNADRKLIGSTAKLIYLSNSLQSITEQSTWTDALKAIGKSMDIDGKVGQY
LAC             QKPSIHFIWGAHNPGNSNNATFKLILLSKSLQSIKGISTYTEAFKSLGKMMDIGDKAIEY
                :***:****    :     :     .     ***  **:***::.   .:::.:*:*::*.  ***....  :*

ORO             ESFCGKLKSLARSSIKKLD-RKIEPIQIGTATVLWEQQFKLDTDVIKREDRIHLMKDYLG
BUN             ETLCAERKMIARSTGKKVDNKRLEAVKIGNALVLWEQQFILANDLFKNQERQKFMKNFFG
LAC             EEFCMSLKSKARSSWKQIMNKKLEPKQINNALVLWEQQFMVNNDLIDKSEKLKLFKNFCG
                *  :*   .  *    ***:  *::     ..:*.   :*..*  *******  :   .*::...:.  :::*::  *
                  L2
ORO             IGKHKSFSKKLNNDINTDKPKILNFNNDDIVRKCKDKYNQVIHNLSQINELDKIGNYLEH
BUN             IGKHKSFKDKTSSDIETDKPKILDFNNTIVLMAARTMVNKNKALLAKDNTLQDLHPIIMQ
LAC             IGKHKQFKNKMLEDLEVSKPKILDFDDANMYLASLTMMEQSKKILSKSNGSKPDNFILNE
                *****.*..*   .*::..*****:*::    :      .       ::       *::  *     .          :

ORO             FSAKISACSVEMWDFIYNTTKTKYWQCINDYSTLMKNMLAVSQYNRHNTFRIVSCANNNV
BUN             YASEIKEASKDTFDALLKISKTCFWQCIVDVSTIMRNILAVSQYNRHNTFRVAMCANDSV
LAC             FGSKIKDANKETYDNMHKIFETRYWQCISDFSTLMKNILSVSQYNRHNTFRIAMCANNNV
                :.::*.  ..  :  :*  :  :    :*  :****  *  **:*.*:*:***********:.  ***:.*

ORO             FGLVMPSSDIKTKKATLVYAIMALHNEEAEIAELGSLYSTFKTAT-GYISISKAFRLDKE
BUN             YALVFPSSDIKTKRATVVFSIVCMHKEKNDLMDAGALFTTLECKNKEYISISKAIRLDKE
LAC             FAIVFPSADIKTKKATVVYSIIVLHKEEENIFNPGCLHGTFKCMN-GYISISRAIRLDKE
                :.:*:**:*****.**:*::*:  :*:*:  ::  :  *.*.  *::    .    *****.*:*****

ORO             RCQRIVSSPGLFLMTSCLLFNGNKSLEFDKLLGFSFFTSISITKAMLSLTEPSRYMIMNS
BUN             RCQRIVSSPGLFILSSMLLYNNNPEVNLVDVLNFTFYTSLSITKSMLSLTEPSRYMIMNS
LAC             RCQRIVSSPGLFLTTCLLFKHDNPTLVMSDIMNFSIYTSLSITKSVLSLTEPARYMIMNS
                ************:  :.  *:  :.*    :  :  .::.*:::**:****::******:*******

ORO             LAVSSHVREYISEKFSPYTKTSFSVVMTDLIKKGCYSAYEQRKKVQIRDIKLTDYDITQK
BUN             LAISSHVRDYIAEKFSPYTKTLFSVYMVNLIKRGCASANEQSSKIQLRNIYLSDYDITQK
LAC             LAISSNVKDYIAEKFSPYTKTLFSVYMTRLIKNACFDAYDQRQRVQLRDIYLSDYDITQK
                **:**:*.:**:*********  ***  *.  ***..*  .*  :*  ..:*:*:*  *:*******

ORO             GVDSKRDLKSIWFPGKVNLKEYLNQIYLPFYFNSKGLHEKHHVLIDLAKTVLEIEKEQRE
BUN             GVNDGRNLDSIWFPGKVNLKEYINQIYLPFYFNAKGLHEKHHVMIDLAKTVLEIEMNQRS
```

```
LAC             GIKDNRELTSIWFPGSVTLKEYLTQIYLPFYFNAKGLHEKHHVMVDLAKTILEIECEQRE
                *:.. *:* ******.*.****:.*********:*********::******:**** :**.

ORO             SLPEPWSEIPAKR-LSLNVLIYSL-QELNLDTSRHNFVRSRVENANNFNRSITTISTFTS
BUN             DNLGIWSKAEKKQHVNLPILIHSIAKSLILDTSRHNHLRNRVESRNNFRRSITTISTFTS
LAC             NIKEIWSTNCTKQTVNLKILIHSLCKNLLADTSRHNHLRNRIENRNNFRRSITTISTFTS
                .    **    *. :.* :**:*: :.*  ******.:*.*:*. ***.***********
                                         L3
ORO             SKSCIKIGDFEEEKREKLRMIQKKLAKDISKLTIANPAFLDEITNEHEIRHSTYEDLKQS
BUN             SKSCIKIGDFREIKDKETEKSKKSTEKFDKKFRLSNPLFLEDEEANLEVQHCNYRALIQK
LAC             SKSCLKIGDFRKEKELQSVKQKKILEVQSRKMRLANPMFVTDEQVCLEVGHCNYEMLRNA
                ****:***** : *  :    :*       *: ::** *: :     *: *..*  * :

ORO             IPDYTDYMSVKVFDRLYEKITTNEINDKETVKLILETMKKHKIFHFGFFNKGQKTAKDRE
BUN             IPNYKDYISVKVFDRLYELLKNGVLTDKPFIELAMEMMKNHKEFSFTFFNKGQKTAKDRE
LAC             MPNYTDYISTKVFDRLYELLDKRVLTDKPVIEQIMDMMVDHKKFYFTFFNKGQKTSKDRE
                :*:*.**:*.******** :  .   :.**  ::  :: * .** * * ********:****

ORO             IFLGEFEAKMCLYLVERIAKERCKLNPEEMISEPGDSKLRVLEKQSEDEIRYISNTIKTL
BUN             IFVGEFEAKMCMYVVERISKERCKLNTDEMISEPGDSKLKILEKKAEEEIRYIVERTK--
LAC             IFVGEYEAKMCMYAVERIAKERCKLNPDEMISEPGDGKLKVLEQKSEQEIRFLVETTRQK
                **:**:*****:* ****:*******.:********.**.:**::*:***:: :  .

ORO             GNAIENLQSGSL---------NWADICENKARGLKIEINADMSKWSAQDVLFKYFWLIVL
BUN             ----DSIIKG-----------------DPSKALKLEINADMSKWSAQDVFYKYFWLIAM
LAC             NREIDEAIEALAAEGYESNLEKIEKLSLGKAKGLKMEINADMSKWSAQDVFYKYFWLIAL
                    :.  ..                 . :..**:**************::******.:

ORO             DPILYPAERKRIIYFLCNYMQKRLIMPDELLTTILDQRVPYSNDIIGLMTNNYRSNTVEI
BUN             DPILYPAEKTRILYFMCNYMQKLLILPDDLIANILDQKRPYNDDLILEMTNGLNYNYVQI
LAC             DPILYPQEKERILYFMCSYMDKELILPDELLFNLLDQKVAYQNDIIATMTNQLNSNTVLI
                ****** *. **:**:*.**:* **:**:*: .:***. .*.:*:*  ***   . * * *

ORO             KRNWLQGNLNYTSSYLHSCSMSVYKDIIREAAILLEGEALVNSMVHSDDNQTSICMVQNK
BUN             KRNWLQGNFNYISSYVHSCAMLVYKDILKECMKLLDGDCLINSMVHSDDNQTSLAIIQNK
LAC             KRNWLQGNFNYTSSYVHSCAMSVYKEILKEAITLLDGSILVNSLVHSDDNQTSITIVQDK
                ********:** ***:***:* ***:*:.*.  **:*. *:**:*********: ::*:*

ORO             LPDDNIIEFCIKIFEKICLTFGNQANMKKTYLTNFIKEFVSLFNIHGEPFSIYGRFLLTA
BUN             VSDQIVIQYAANTFESVCLTFGCQANMKKTYITHTCKEFVSLFNLHGEPLSVFGRFLLPS
LAC             MENDKIIDFAMKEFERACLTFGCQANMKKTYVTNCIKEFVSLFNLYGEPFSIYGRFLLTS
                : :: :*::. : **  ***** ********:*:  ********::***:*::*****.:

ORO             VGDCAYLGPYEDLASRLSATQTAIKHGCPPSLAWVSIALNHWITHTTYNMLPGQNNDPLP
BUN             VGDCAYIGPYEDLASRLSAAQQSLKHGCPPSLVWLAISCSHWITFFTYNMLDDQINAPQQ
LAC             VGDCAYIGPYEDLASRISSAQTAIKHGCPPSLAWVSIAISHWMTSLTYNMLPGQSNDPID
                ******:*********:*::* ::********.*::*: .**:*  ***** .* * *

ORO             FFPTNNRSEIPVEMCGILESDLSTIALTGLEAGNVTFLTNIARKLSSPILQRESIQDQYN
BUN             HLPFNNRKEIPVELNGYLNAPLYLIALVGLEAGNLWFLINILKKLVPLDKQKETIQSQCL
LAC             YFPAENRKDIPIELNGVLDAPLSMISTVGLESGNLYFLIKLLSKYTPVMQKRESVVNQIA
                .:* :**.:**:*: * *:: *  *: .***:**: ** ::  *  .   :.*:: .*

ORO             SIEKWDLSKLSQIDILRLKMLRYISLDSSVTSDDGMGETSEMRSRSLLTPRKFTTSGSLN
BUN             HLCN-SIDKLTESEKFKLKILRYLTLDTEMSVDNNMGETSDMRSRSLLTPRKFTTLGSLN
LAC             EVKNWKVEDLTDNEIFRLKILRYLVLDAEMDPSDIMGETSDMRGRSILTPRKFTTAGSLR
                 : : .:..*:: : :.**:***: **:.:  .: *****:**.**:******** ***.

ORO             RLKSYKDFQDIIADEDKTNELFENFIRHPELLVTKGETFEEFVNTILFRYNSKKFKESLS
BUN             KLVSYNDFRSSL-DDQRFTDNLNFMLNNPELLVTKGENKEQFMQSVLFRYNSKRFKESLS
LAC             KLYSFSKYQDRLSSPGGMVELFTYLLEKPELLVTKGEDMKDYMESVIFRYNSKRFKESLS
                .* *:..:.. : .     : :  :: :*********  :::::::******.******

ORO             IQNPAQLFIEQILFSNKPVIDYTSIHDKIFGLQDMPGIEELDTIIGRKTFVESYVQIVDD
BUN             IQNPAQLFIEQILFSHKPIIDYSSIFDKLTSLAEADIIEELPEIIGRVTFPQAYQMINRD
```

```
LAC                IQNPAQLFIEQILFSHKPIIDFSGIRDKYINLHDSRALEKEPDILGKVTFTEAYRLLMRD
                   ***************:**:**::.* **  .* :   :*:    *:*. ** ::*  :  *

ORO                LSNLTLDINDVKTIFAFCLMNDPLLITSANNIIMSVKGHSQERIGQSACKMPEVRSLKLI
BUN                IGQLPLDIDDIKLIFRYCILNDPLMITAANTSLLCVKGTPQDRTGLSASQMPEFRNMKLI
LAC                LSSLELTNDDIQVIYSYIILNDPMMITIANTHILSIYGSPQRRMGMSCSTMPEFRNLKLI
                   :..* *  :*:: *: : ::***::** **. ::.: * .* * * *.. ***.*.:***

ORO                HYSPAVVLRAYVRGPTNVPNVDIDELARDLSHLEDFIQSTKLRENMRERIEINEKRHL--
BUN                HHSPALVLKAFSKGTSDIPGADPIELEKDLHHLNEFVETTAIKEKILHNID-NPPKHLIG
LAC                HHSPALVLRAYSKNNPDIQGADPTEMARDLVHLKEFVENTNLEEKMKVRIAINEAEK--G
                   *:***:**.*: .. .:: ..*  *: .** **::*::.* : *::  .*  *    :

ORO                GRDFKFEIKELTRFYQVCYDYIKSTEHKVKVFILPYKVFTSIEFCGALTGNLINDKLWYI
BUN                NEILIYRIREMTKLYQVCYDYVKSTEHKVKIFILPMKSYTAIDFCTLIQGNTISDNKWYT
LAC                QRDIVFELKEMTRFYQVCYEYVKSTEHKIKVFILPTKSYTTTDFCSLMQGNLIKDKEWYT
                      : : :.*:*.:*****:*:******:*:**** * :*: :**  : ** *.*: **

ORO                THYLKNIVSTTHKAQISSSPELELQIADEALRLVAHFADTFLASESRIQFLKKIIEEFTY
BUN                MHYLKQIASGSIKGNIVTTSTSEQIIANECFRVLCHFADSFVEEASRLSFINEVLDNFTY
LAC                VHYLKQILSGGHKAIMQHNATSEQNIAFECFKLITHFADSFIDSLSRSAFLQLIIDEFSY
                    ****:* *   *. :  ..  *  ** *.:.:: ****:*: . **  *:: ::::*:*

ORO                KGIPVKHLYSKIKNSKLRVKFLGILLWLDDLTQNDLDKFDADKSDEKIIWNNWQVSRDMN
BUN                KNISVNSLFNTLLASTTRLDFIPLLFRLKVLTQTDLNRFDALKTNERVSWNNWQTNRSLN
LAC                KDVKVSKLYDIIKNGYNRTDFIPLLFRTGDLRQADLDKYDAMKSHERVTWNDWQTSRHLD
                   *.: *. *:. :   .  * .*: :*:.    * * **:.:** *: *.: **:**..* ::

ORO                TGPIDLMISGYSRQLRITGEDDKLIAAELQVTRLSEDLIYRHGQAMLNKPHGLKLEKMQP
BUN                SGLIDLTISGYLRSIRVVGEDNKLKIAELTIPNFYPNTVFHAGNKLLNSRHGLKFEYMEE
LAC                MGSINLTITGYNRSITIIGEDNKLTYAELCLTRKTPENITISGRKLLGARHGLKFENMSK
                    * *:* *:** *.: : ***:**  *** :..   : :   *. :*.  ****:* *.
                                                                L4
ORO                VTEMSKRLHYIVFQQRSRKRYFYSILPTQVIEDHNSRVESSRLSRDSKWVPVCPVAISKL
BUN                IVLDEKYNYYITYQKKRAHIYTYQVSTIEHILRRNNEGLQSRGPRYNKMVPVCPVVLSVR
LAC                I-QTYPGNYYITYRKKDRHQFVYQIHSHESITRRNEEHMAIRTRIYNEITPVCVVNVAEV
                   :       :**.:.:.  : : *.: . : *  .*.      *     .: .*** * ::

ORO                YQQGRPILSKVRNLNMQTHSLSRIQVNVDEYAITRRAHFQKMPFFEGPSIPSGGMDLSEL
BUN                DELFRMSLENVFSLNMTNFSMSRLFVSPDEVATVKKAHMSKMMFFSGPTIKAGIINLTSL
LAC                DGDQRILTRSLDYLNNDIFSLSRIKVGLDEFATIKKAHFSKMVSFEGPPIKTGLLDLTEL
                       *    .:  **   .*:**: *. ** *   ..**:.**  *.**.* :* ::*:.*
                                                         L5
ORO                MKSTSLLSLNYDNIKNASLLDMSRVFKCNGSGDDQMAFEFLSDEILEQDVVEEIECNPIF
BUN                MRTQELLTLNYDNLCKSSIVPFCRILECNG--DEQGELIFLSDEVMDFTISEEIESMPLF
LAC                MRSQDLLNLNYDNIRNSNLISFSKLICCEGSDNINDGLEFLSDDPMNFTEGEAIHSTPIF
                   *.: .**.*****: ::.:: :..:: *:*   : :   : ****: ::     * *  . *:*

ORO                SISYTKRGESNMTYKNAFHKALISECDKFEEAFDFLDMGFCSNENLSILEEIHWIISYLK
BUN                TIRYQKRGTEIMTYKNAIMKLVSAGVDEIKEVFDFSKQGFYSKKNLGIINTICSIINILE
LAC                NIYYSKRGERHMTYRNAIKLLIERETKIFEEAFTFSENGFISPENLGCLEAVVSLIKLLK
                   .* * ***   ***.**:    :     . ::*.* * . ** * :**. :: :  :*. *:

ORO                TNQWSTELDNCIHMCMYRNGYDAEYHKFDIPSKFLKDPINRTINWTEVIEFILLIEDFQT
BUN                TNEWSTILYNSFHIAMLLESMDREFHMFTLPEAFFINVAGGVVNWTKLLKFIKSLPV--I
LAC                TNEWSTVIDKCIHICLIKNGMDHMYHSFDVPKCFMGNPITRDINWMMFREFINSLPG--T
                   **:*** : :.:*:.:  :. *  :* * :*. *: :      :**  . :**  :

ORO                KIEPWSSMKSHFCSKAHSVALECMKNEKRSLAEFVDKSKKT-GKSKFDF-
BUN                EQEPWSMMMSRFVEKTVYL-IEREMNKDVDFTDFLDELEFSSGKSLFTFF
LAC                DIPPWNVMTENFKKKCIAL-INSKLETQRDFSEFTKLMKKEGGRSNIEFD
                   .  **. * ..* .*   : ::   : . .:::* .  :   *.* : *
```

The alignment was produced using MUSCLE [Edgar R.C. (2004) MUSCLE: multiple sequence alignment with high accuracy and high throughput. Nucleic Acids Research 32(5): 1792-1797.];

*, indicates identical amino acid, :, indicates similar amino acid residues. Positions of insertion of the V5 epitope are shown in red.

(b) SCRATCH prediction:

```
1                                                          60
MEDQAYDQYLHRIQAARTATVAKDISADILEARHDYFGRELCNSLGIEYKNNVLLDEIIL
CCHHHHHHHHHHHHHHHHHHHHHHCCCHHHHHHHHHHHHHHHHHHHCCCEECCCCCHHHHEE
.HHHHHHHHHHHHHHHHHHHHHHHHHHHHHHHHHHHHHHHHHHHHHHT.EEET.EEHHHHHE
----++++++++++++--+-++----+++++++++--+++++++------------++++
---------------++-+-+-+----+--+-+++--+---++----++++----+++++
-----------+---++-+--+++---+-++-++----++++-----++++---++++++
-----------+---------------+--+-+------+++-----+-+---+++++++
eeeeeeeee-ee-eeeeeee-eee-eee--eeeeeee-ee--eeeeeeeeee---ee---
eeeeeeee--ee-ee-ee-e--ee--ee--eee-ee--ee--ee---e-eee---ee---
eeee--ee--ee-ee-ee-e--ee--ee--eee-ee--ee--e----e-e------e---
eeee--ee--ee-ee-ee-e--ee---e--e---e---ee--e----e-e----------
eeee---e--------------e-----------e-------------------------


61                                                        120
DVVPGVNLLNYNIPNVTPDNYIWDGHFLIILDYKVSVGNDSSEITYKKYTSLILPVMSEL
ECCCCCCCCCECCCCCCCCCEEECCCEEEEEEEEEEEECCCCCEEEHHHHHHHHHHHHHC
EE.TTEEEEEE.......T.EEETSEEEEEEEEEEEE.....HEEHHHHHEHHHHHHHHT
-------------------++++--++++++++-++--------++-+++----++++--
++-+--+--+-+-----+-++++--++++++++++++-+-----+-----------+---
++----+++--+-----++++++--++++++++++++-++-++-++-+++----------
++----+----+-----+-++-+-++++++++++++-----++-+--++--+--------
e--ee-e--eee-eeeeeeee--e-e-------e-eeeeeeee-eeeeeeee--e--eee
---e--e--e-e-ee-eeee---e---------e---eeeeee-e-ee------e--ee-
---e-------e-ee-e-e--------------e---eeeeee-e-ee---------ee-
--------------e---e--------------e---eeeeee---ee---------ee-
--------------e-----------------------ee--e---ee------------


                            L1

121                                                       180
GIDTEIAIIRANPVTYQISIIGEEFKQRFPNIPIQLDFGRFFELRKMLLDKFADDEEFLM
CCCEEEEEEEECCCCEEEEECCHHHHHHCCCCCEEECCHHHHHHHHHHHHHHCCHHHHHH
T..EEEEEEEES.EEEEEEEEHHHHHHH.TT..EEE.HHHHHHHHHHHHHHH..HHHHHH
-----+++++--------+--+--++-------------+++++++++++-++---++++
---++++++++++---+++------+------------------+---------------
--+++++++++++-----+-----------------+--+----+---------------
---+++++++++----+++---------------+-+--+--+++--+----------++
eeeee----e-ee-eee-e--eee-eeeeee-e-e-eeee--e-eee--ee-eeeee---
e-eee----e-ee-e-e-----ee-eee-ee-e-e-e--e--e--e---ee--eeee---
e-e-e----e--e---e-----ee-eee-ee-e-e----e--e--e---ee--eee----
--e---------e---------ee-eee-ee---e----------e---ee--eee----
----------------------e--ee--ee--------------e----e--e------


181                                                       240
MIAHGDFTLTAPWCTSDTPELEEHEIFQEFINSMPPRFVSLFKEAVNFSAYSSERWNTFL
HHHCCCCEECCCCCCCCCCCHHHCHHHHHHHHCCCCCHHHHHHHHHCCCCCCCHHHHHHH
HHHTT..EEE......S.HHHHHHHHHHHHHHTS.HHHHHHHHHHHHHHHH.HHHHHHHH
++++-------------------+-++++++-------+-++++++---------+++++
+-------------------------------------------------------+---
--------------------------------------------+---------------
+---------------------------+-------------+-+--------------+
```

```
---eee-e-e-ee-eeeeeeeeeee--ee--eeeeee-eee-ee-eeeeeeeeeeeee--
---e-e-e-e-e--eeeeeeeeeee--ee--ee-eee--e--ee--e-eeeeeee-ee--
---e-e-e-e----eeeeee-eeee--ee--ee-eee--e--ee--e-eeeeeee-e---
---e-e-e-e----eeeeee-ee-e--ee--ee-eee--e--ee--e-e---eee-----
---------------ee-ee-ee-e------ee--ee-----ee--e------ee-----

241                                                      300
YRARAETEVDYNQFLSDKAHKIFMLEGDYMRPTQAEIDKGWELMSQRVYTEREIITDVTK
HHHHHHHHHHHHHHHHHHHHHEEEECCCCCCCCHHHHHCCHHHECCCCCCHHEHHHCHHC
HHHHHHHHE.HHHHHHHHHHHHHHHTT.T....HHEE.THHHHHHHEEEEHHEEEEE...
++++--+--++-+--+--+++++++----------++---+-----------+++-----
++++--------------++++++-------------------+----------------
-+++--------------++++++-+-----------------+----------------
++-+--------------+++----+---------------+-+----------------
ee-eeeeeeeeeee-eee-ee--e-eeeeeeeeeee-eeeeeeeeeeeeeeeeeeeeeee
-e-eeeeeee-ee--eee--e----eeeeeeeeeee-eee-e--eee-eeeee--eeeee
-e-e-eee-e-ee---ee--e----e-e--eeeeee-ee--e---ee--eeee--ee-ee
-e-e-e-e-e--e---ee--e------e--e--eee-ee--e---ee--eeee--ee-ee
-------e--------ee------------e------ee--e--------------e-ee

301                                                      360
QKPSIHFIWVKNADRKLIGSTAKLIYLSNSLQSITEQSTWTDALKAIGKSMDIDGKVGQY
CCCCEEEEEEECCCCCCCCHHHHHHHHHHCHHCCCCCCHHHHHHHHHCCCCCCCCCEEEH
....EEEEEEEHHHHHHHHHHHHHHHHHHHHHHHHHH.HHHHHHHHHTH....TS.E.HH
-----++++++--------+++++++++-----------+++++++++--------+--+
--++++++++++--------++++++++--------------+-+---+---------+-
----+++++++---------+++++++++----------+-++++---+-----------
---++++++++---------+++++++++++-----------+++--+----------+-
eeee-e----eeeeeeeeeeeee--e-eeeeeeeeeeeeeee--eeeeeeeeeeeeeeee
eeee------ee-eeeeeeee-e--e--ee-ee-eeeee-ee--ee-eee-e-eeeeeee
eeee------ee-eee--eee-e-----ee-ee-eeeee-ee--ee-eee-e-eeeeee-
eeee------ee-eee--e---e-----e--ee-eee-e-ee--ee-eee-e-eee--e-
ee-------------e------------e--ee-eee-e--e--ee--e--e-e-e----

361                                                      420
ETLCAERKMIARSTGKKVDNKRLEAVKIGNALVLWEQQFILANDLFKNQERQKFMKNFFG
HHHHHHHHHHHHCCCCCCCCCCCCHHHHHHHHHHHHHHHHHHHHHHHCHHHHHHHHHHCC
HHHHHHHHHHHHHTS....THHHHHHHHHHHHHHHHHHHHHHHHHHHHHHHHHHHHHHTT
++++++++++++------------+----+++++++++++++++++----+++++++++-
+------+---------------------++-++-+++------------+---+++---
+------+---------------------+---++++++-+++-------+---++----
+------+---------------+--+--+++++++++++++++----------+++---
ee--eeeeeeeeeeeeeeeeeeeeeeeeee-----eee-e--eeeeeeeeeeeeeee-ee
ee--eeeee-eeeeeeeeeeee-e--e-ee-----ee-----ee--eeeeeee--ee---
ee--eeee---eeeeeeeeeee-e--e--e-----e------ee--eeeeeee--ee---
e---e--e---eeeeee-eeee-e--e--e------------ee--eeeeeee--ee---
-------e---e-eeee-eee--e--e-------------------eeee--e--e----

       L2

421                                                      480
IGKHKSFKDKTSSDIETDKPKILDFNNTIVLMAARTMVNKNKALLAKDNTLQDLHPIIMQ
CCCCCCCCCCCCCHCCCCCCCECCCHHHHHHHHHHHHHHHHHHHHHHHCCCCCCCHHHHH
E.................S.EEE..HHHHHHHHHHHHHHHHHHHHHHH.HHHHHHHHHHH
--------------------------+++++++++++++++++++++--------+-+++
-------+---------------+--+++++++++-+--+++---++------------+
-------+------------+-++--+++++++++++---++-++++-------++-+++
-------+-----+-+-------+--++++++++++++++++--++--------+--+++
eeeeeeeeeeeeeeeeeeeeeeee-ee--------eeeeeeeeeeeeeee-ee-eee-ee
-eeeee-eeeeeeeeeeeeee--e-e--------e---eeeee--eeeee-ee--e---e
```

```
-eeeee-eeeeeee-eeeeee--e-e--------e---eeeee--eeeee-ee--e---e
-eeeee-eeeeeee-eeeeee--e-e------------ee-e---eeeee-ee--e---e
--e-e--eeeeeee-eeeeee------------------e-----ee-e--ee-------


481                                                      540
YASEIKEASKDTFDALLKISKTCFWQCIVDVSTIMRNILAVSQYNRHNTFRVAMCANDSV
HHHHCCCCCCCHHHHHHHHHHHHHHHHHECHHHHHHHHHHHHHHCCCCCEEEEEECCCCE
HHHHHHHH.HHHHHHHHHHHHHHHHHHHHHHHHHHHHHHHHHHHHTT..EEEEEEHTS.E
+++--------+++++++++++++++++++++++++++++++++-----+++++++--++
-+------------+++-++--++--++++++--+++++++---+----+++++++++++
++-------------++-++++++++++++++--+++++++++-----++++++++++++
++-------------++-++++++++++++++--+++++++++-----++++++++++++
e-ee-eeeeeee-ee-eeeeee-eee---e-eeeeee-e--eeeeeeee-e----eeee-
--ee-eeeeeee-ee--e-eee---e---e--e--e------eeeeeee-e-----ee--
--ee-ee-eeee-ee--e-eee---e---e--e--e------e-eeeee-e-----ee--
--ee-ee-eeee-e---e--ee----------e--e------e-eeee--------ee--
--ee-ee--ee--e---e--e------------------------ee---------e---


541                                                      600
YALVFPSSDIKTKRATVVFSIVCMHKEKNDLMDAGALFTTLECKNKEYISISKAIRLDKE
EEEEECCCCCCCCCCEEEEEEEEEEECCCCCCCCCCCCCEEEECCCEEEEECHHHHHCHH
EEEE...S.....EEEEEEEEEEE.H.H.HHHHHHHHHHEHEE.THEEEEHHHHHHH.HH
++++++--------+--++++++++----------+--++++-----+++++++++++++
+++++++------+++++++++++++----+----+--+++++---++++++++---++-
+++++++---+--+++++++++++++---------+++++++++--+++++++++++++-
+++++++---+---++++++++++++---------+++++++-+--+++++++++++++-
e---eeeeeeeeee-e-------eeeeeeeeeeeeeeeeeee-eeeeeee-eeeeeeeee
-----eeeeeeeee-e--------eeeeee-eeee---ee-e-eeeee-e-ee--eeeee
-----eeee-eeee----------eeeeee-ee-e---e--e-eeeee-e-ee--eeeee
------eee-eeee-----------eeeee-ee-e------e-eeee--e--e--e-eee
-------ee-eeee-----------eeee---e----------eeee-----e--e--ee


601                                                      660
RCQRIVSSPGLFILSSMLLYNNNPEVNLVDVLNFTFYTSLSITKSMLSLTEPSRYMIMNS
HHHHHHHCCCCEEEEEHHHCCCCCCCCHHHHHHHHHHHHHHHHHCEEEECCCCHCCCCCC
HHHHHH.SHHHHHHHHHHHHTT.T.EEEEEEEEEEEEEEHHHHHHHHH.....HHHHHHH
++++++------++++++++---------++-++++++++++++++++-+--++++++++
+++-+-----++++++++--------+--++-+++++++++++++++-+--+++++++--
+++-++----++++++++---------+-++++++++++++-++++++++++++++++++
+++++++++-++++++++---------+-+++++++++++++++++++++++++++++++
e-eeeeeeee---------eeeeee-e--e--e-e-eee-e-eeee-eeeeeeee---ee
e-ee--eee-----------eeeee-e--e--e-------e--ee--e-eee-e------
e-ee--eee-----------eeeee-e-----e-------e--ee--e-eee-e------
e-ee--e-e------------eeee------------------e---e-eee--------
--ee--------------------e------------------e----------------
◘

661                                                      720
LAISSHVRDYIAEKFSPYTKTLFSVYMVNLIKRGCASANEQSSKIQLRNIYLSDYDITQK
HHHCCCHHHHHHHHCCCCCHHHHHHHHHHHHHHHHHHHHHCCCCECECCEEEECCCCCCC
HHHHHHHHHHHHHH..HHHHHHHHHHHHHHHHTT...HHHH.HHEEEEEEEEE...EEEE
-+-++-+++++++-------+++++++++++++++++++---------------------
-------+--+--------+++++++++++++++++++---+---++-++++++-+-+--
++----++--+--+----+++++++++++++++++++++----+-+++++++++------
+++++-++++++------++++++++++++++++++++----++-+++++++++------
e--eee-eee-eeeeeeeeee----e--ee-eee-eeeeeeeeeee-ee-e-eeee-eee
----ee-eee--ee-eeeeee-------e--eee-eeeeeeeee-e-ee-e--eee-eee
----ee-ee---ee-eeeeee-------e--ee--eeeeeeeee-e-e-----e-e-eee
----ee--e---ee--e--e-----------ee--ee-eeeeee-e-e-----e-e-eee
------------ee--e--e-----------ee--ee-eee--e---------------e
```

```
721                                                      780
GVNDGRNLDSIWFPGKVNLKEYINQIYLPFYFNAKGLHEKHHVMIDLAKTVLEIEMNQRS
CCCCCCCCCCCCCCCHHHHHHHHHHHHHHHHHCHCCHHHHHHHHHHHHHHHHHHHHHHHH
...TT....EEE.TT.E.HHHHHHHHE.HEEEEHTTHHHHHHHHHHHHHHHHHHHHHHH.
++-------+---+---+++++++++++++++++---+--++++++++++++++++++++
++-----+++++++---+++--++++++++++++---+---+--+--++-+-+-+---+-
++++---+++++++++-+++-+++++++++++++---+--++-++--++-+++++-+-+-
++-+---+++++++++-+++++++++++++++++------++++++-++-+++++-+-+-
eeeeeeeeee-e-eeeee-eee-ee-e-e-e-e-eeeeeeee---e--ee--e-eeeeee
e-eeeee-e----e-eee-ee--ee---------ee-eee-----e--e---e-e-eeee
e-eeeee-e----e-e-e-ee-------------ee-eee-----e--e---e-e-eeee
--eeeee------e-e---ee-------------e---ee-----e--e---e---eee-
--ee-e---------e----e-------------e---ee--------e--------ee-
```

```
781                                                      840
DNLGIWSKAEKKQHVNLPILIHSIAKSLILDTSRHNHLRNRVESRNNFRRSITTISTFTS
HHHCHHHHHCHCCCCCHHHHHHHHHHHHHHCHHHHHHHHHHHHCHHHCCCCHHHHHHCCC
SHHHHHHHHHHHH...HHHHHHHHHHHHHH...THTTHH.EEE..TT..EEEEEEEEEE.
+++++++---------+++++++++++++-++--++++++++-+--+------++++---
-----------------+++++++++----+------++---------------------
---------------+-+++++++++----+--+---++-+---+--++++--+---+--
---------------+++++++++++-+--+--+---++-+---------+--+-+-+--
eeeeeeeeeeeeeeee-e---ee--ee--eeeeeeee-eeeeeeeeeeeeeeeeeeeeee
ee-e--eeeeeeee-e-e---e---e---eeeeeeee-eee-eeeee-eee-ee-eeeee
ee----eeeeeeee-e---------e----eeeeeee-eee-eeeee-ee--ee-ee-ee
ee-----eeeeeee-e---------e----e-eeeee-eee-eeeee-ee--ee--e-ee
ee-----e-eeee--e----------------ee-e--ee--eeeee-ee------e-ee
```

L3

```
841                                                      900
SKSCIKIGDFREIKDKETEKSKKSTEKFDKKFRLSNPLFLEDEEANLEVQHCNYRALIQK
CCHHHCCCCCCHCCCHHHHCCCCCCCCCCCEEEECCCCCCCCCEEEEEHCHHHHHHHHCC
.SEEEEEE.EHEEE.HH........HHH.TEEEE.SHH...HHHHHHHHHHHHHHHHHHH
----+-+-----------+--------------+----+-----+++-----++++++--
--+++-------------------------++-++--------+++++++++++++++--
++++++------------------------+-++----++--+-++++--++++++++--
-+++++-+-------------------------+------++++++++--+++++++---
eee-eeeeeeeeeeeeeeeeeeeeeeeeeeeeeeeeeeeeeeeeeeeeeeeeeeee--ee
eee--e-ee-ee-eeeeeeeeeeeeeeeeee-e-eeee-eeeeeeeeeeee-e-ee--ee
eee--e-ee-ee-eeeeeeeeeeeeee-eee-e-eee---eeeeee-e-ee-e-ee--ee
eee--e-ee-ee-eeeeeeeeeeeeee-eee-e-e-----eeee-e-e-e--e-e---ee
ee---e----ee-eeeeeeeeeeeeee-eee-e-------eeee-e-e----------ee
■
```

```
901                                                      960
IPNYKDYISVKVFDRLYELLKNGVLTDKPFIELAMEMMKNHKEFSFTFFNKGQKTAKDRE
CCCCCEEEEHHHHHHHHHHHHCCCCCHHHHHHHHHHHHHCCHHHEEHHEECCCCCHHCCC
.TT...EEEHHHHHHHHHHHHTT....HHHHHHHHHHHHHTHEEEEEEE.TT...HHHHH
-----------++++++++++-------+++++++++++----+++++++-+--------
-------+++-+----+--------------+-++------------+------------
--------+++-+--+-------------+++-++---------+++++---------+-
------+-+++++-+++-----------++++-++--+-----+++++++--------+-
eeeeeee-e-e--ee-ee--eeeeeeeee--e--eeeeeeeee-e-e-eeeeeeeeeeee
-eeeeee---e--ee--e--ee-e-eeee--e---e--eeeee-e-e--eeeeeeeeeee
-ee-ee----e--ee--e--ee---eeee--e---e--ee-ee---e--eeeeee-eeee
-ee-ee----e--e---e--ee---eeee--e---e--ee-ee------eeeeee-eeee
-ee-e--------e---e--ee----ee-------e--ee-ee------eeeee--ee--
```

```
961                                                      1020
IFVGEFEAKMCMYVVERISKERCKLNTDEMISEPGDSKLKILEKKAEEEIRYIVERTKDS
EEEECCCHHHHHHHHHHHHHHHHHCCCHHHHCCCCHHHHHHHHHHHHHHHHHHHCCCHHH
HHHHHHHHHHHHHHHHHHHHHHHH...HHHH..TT.HHHHHHHHHHHHHHHHHHHH..HH
+++----++++++++++++++++------++------+++++++++++++++++-----+
--------+++++++++-----+------++------++--++--++-+++-++------
-+++----+++++++++++---++-----+++-----+++-++--++-+++-++------
-++++-+-+++++++++++---++--+-++++-----+++-++--++-+++-++------
eeeeeeeee--ee--eeeeeee-eeeeeeeeeeeeeeeeeeeeee-eee-ee--eeeeee
----e-eee---e--ee-eeee-eeeeeee-eeeeeee-ee-eee-eee-ee--eeeeee
----e-e-e------ee-eeee-eeeeeee-eeeeeee-e--eee-eee-e---eeeeee
----e-e-e------ee-eeee-eeeeee--eeeeeee-e--eee-eee-e---ee-ee-
---------------e---ee--e---ee--eeeeeee-e--eee-ee------e--ee-

1021                                                     1080
IIKGDPSKALKLEINADMSKWSAQDVFYKYFWLIAMDPILYPAEKTRILYFMCNYMQKLL
HHCCCCCHCEEEEEECCCCCCCCCCCCHHEEEHHHCCCCCCCHHHHHHHHHHHHHHHCCE
HEES.HHHHEEEEEEHT..HHHHHHHHHHHHHHHHH.HT..HHHHHHHHHHHHHHHHHHH
-------++++++--+----------+++++++++-+------+++++++++++++++--
-------+-++++++-+--------++++++++++++-------+++++++++-++++++
-------+-+++++++++-+-----+++++++++++++------++++++++++++++++
-------++++++++++--+-+--++++++++++++++------++++++++++++++++
eeeeeeeee-eee-eeeeeeeeeee---e-------ee--ee-ee-e------ee-ee--
--eeeeeee-eee-eee-eeee-ee---e------------e-ee-e------e---e--
--eeeeeee-eee-eee-ee-e-e-------------------ee--------e---e--
--eeeeee--e-e-eee-ee---e-------------------ee------------e--
--eeeeee--e----ee-ee-----------------------e----------------

1081                                                     1140
ILPDDLIANILDQKRPYNDDLILEMTNGLNYNYVQIKRNWLQGNFNYISSYVHSCAMLVY
ECCCHHHHHHHHHCCCCCHHHHHHHCCCCCCCCEEECCCHHHCCHHHHHHHHHHHHHHHH
H..HHHHHHHHHT.....HHHHHHHHTT..HHHHEEEHHHHTT.EEEEHHHHHHHHHHHH
--+--+++++++--------+++++-------------++-++++-++++++++++++++
+++-+-+++++--+------++--+------++++++++++++++++++++++++++++-
++++++++++++--------++-++------+++++++++++++++++++++++++++++
++++++++++++--------++--+------+++++++++++++++++++++++++++++
---ee---e--eeeeeeeee---e-eeeeeeee-e-eeeeeeee-ee-eee-ee---e--
---ee------eeeeeeeee---e--ee-e----e-eee--eee-e----e---------
---ee------eeeee-eee------ee--------eee--e-e-e--------------
---ee-------eeee--ee------e--------------e---e--------------
-------------e-e----------e--------------e------------------

1141                                                     1200
KDILKECMKLLDGDCLINSMVHSDDNQTSLAIIQNKVSDQIVIQYAANTFESVCLTFGCQ
HHHHHHHCHHCCCCEEEEEEEECCCCCCHHHHHHCCCHHHHHHHHHHHHHHHHEHHEEEE
HHHHHHHHHHHTT.EEEE.EEE..STHHHHHHHHHHHHHHHHHHHHHHHHHHHHHHHHHH
+++++-++-----+++++++-++---+++++++----++++++++++-+++++++++++-
+-+--------+-+++++++++++++++++++++----++-++--+----++++++++++
++++-------+++++++++++++----++++++-------++-++++++++++++++++
++++--+----++++++++++++++---++++++----+--+++++++++++++++++++
ee--ee--eeeeee---e-eeeeeeeeeeee--eeeeeee---ee-eeeeee--eeee-e
ee--ee--ee-eee---e---eeeeeee-----eee-eee---e---ee-ee---e-e-e
ee--ee--e--eee---e---eeeeeee-----eee-eee---e---ee-e----e---e
ee--ee--e--e-e-------eeeeeee-----eee-eee-------ee-e--------e
----ee--e--e-----------eee---------e---e-------e--e---------

1201                                                     1260
```

ANMKKTYITHTCKEFVSLFNLHGEPLSVFGRFLLPSVGDCAYIGPYEDLASRLSAAQQSL
ECCCCCEECCCCHHHHHHECCCCCHHHHHHHHHHCCCCCCCCCCHHHHHHHHHHHHHHHH
HHHHEEEEEE.HHHHHHHHHHTT...EEHEEEE......EEEE..HHHHHHHHHHHHHHH
------------++++++++----++++++++++-+---------+++++++++++++++
+-+--------+-++++-----++++++++++++++--+-------+++--++-+++-++
+++-+----+-++++++++-+++++++++++++++++++----+++++++-+++++++++
+++-+----+-+++++++++++++++++++++++++++++---+++++++++++++++++
eeeeeeeeeee-ee--e--eeeeeeee-e-e-e-eeeee--eeeeeee--ee-eeeeeee
eeeeeee-eee-ee--e--e-eeee-----e---eeeee--e--e-ee--ee-ee-eeee
ee-eeee-eee-ee--e--e-eeee-----e----e--e-----e-ee--ee-ee-eee-
ee-eeee-ee--ee--e--e-eeee-------------e-------ee--ee-ee-ee--
-e-ee-------ee-------eee----------------------e---e--ee--e--

1261 1320
KHGCPPSLVWLAISCSHWITFFTYNMLDDQINAPQQHLPFNNRKEIPVELNGYLNAPLYL
HCCCCHHHHHHHHHHHHHHHHHHHCCCCCCCCCHHHHCCCCCCCCCCCCCCCCCCCCHHH
HTT....EEEEEEE.HHHHHHHHHHHHHHH....GG....T......EEETT..THHHHH
-------+++++++++++++++-++--------+++-+------------------++++
-------++++++++++++++-+++------+++----+-------+----+++-+++++
----++++++++++++++++++++++-----+++--+++-------++-+++++++++++
----++++++++++++++++++++++-----++++-+++-------++++++++++++++
eeeeeeee-e---e-eee-----eee-eeeeeeeeeeeeeeeeee-eee-eee-e-----
eee-eee--------ee-------e--eee-eeeeee-eeeeeee-eee-e-e-e-----
eee-eee--------e--------e--eee-e-eeee-e-eeeee-e-e-e---------
eee-eee--------------------eee-e-eeee-e-eeeee---e-----------
eee-ee----------------------e--e---e--e-e--ee---e-----------

1321 1380
IALVGLEAGNLWFLINILKKLVPLDKQKETIQSQCLHLCNSIDKLTESEKFKLKILRYLT
HHHHCCCCHHHHHHHHHHHHHCCHHHHHHHHHHHHHHHHCCHHHCCCCHHHHHHHHHHEC
HHHHHHHHHHHHHHHHHHHHH..HHHHHHHHHHHHHHHHHHHHHH.HHHHHHHHHHEEEE
+++++----++++++++++-+-----++++++++++++++-+++--+++++++++++-
++++++---++++++++-+--------+---+-++-------------+++++++-++++
+++++++++++++++++++--------+--+++++--++---------++++++++++++
+++++++++++++++++++-+------+--++++++-++---------++++++++++++
------e-ee-----e--eeeeeeeeeeeeeeee-eee-eeeeeeeeeeeee-e--ee-e
---------------e--ee--eeeeeeee-eee-ee--eeeeeeeeeee-e-e--e--e
------------------ee--eeeeeeee-eee-ee--ee-eeeeeeee-e-e--e---
------------------ee--eeeeeeee-eee-ee--ee-ee-eeeee-e-e--e---
------------------ee--e-eeeeee-e----e--e--ee-ee-ee----------
◘

1381 1440
LDTEMSVDNNMGETSDMRSRSLLTPRKFTTLGSLNKLVSYNDFRSSLDDQRFTDNLNFML
CCCCCCCCCCCCCHHHHHEEEECCCCCCCCHHHHHHHCCHHHHHHHCCCHCHHHHHHHHH
E..EEEE.TTT......H........EE.EHH.HHHHHEHHHHH...HHHHHHHHHHHHH
-----------++++++++++-----------+++------++++-+---++++++++++
+---------------+----+-++------------------+--------------+-
+-----------++--+--+-+++++-------+----+----+-------+---++-+-
+-----------++++++++++++++---------+--+----+----------++++++
eeeeeeeeeeeeeeeeeeeeeeeeeeeeeeeeeeeeeeeeeeeeeeeeeee-eee-e-e-
-eee-e-eeeeeeeeeeeeeeeeeeee-eeeee-ee--e-ee-eee-eeee-eee-e---
-eee-e-eeeeeeeeeeeee-e-eeee-ee-ee-ee--e-ee-eee-eeee-eee-----
-eee---eee-eeeee-e-e---eeee-ee-ee-ee----ee-eee-eeee--e------
---e----ee-ee--e-e-------ee--------e----e------ee----e------

1441 1500
NNPELLVTKGENKEQFMQSVLFRYNSKRFKESLSIQNPAQLFIEQILFSHKPIIDYSSIF
CCCCEEEECCCCHHHHHHHHHHHCCCHHHHHHHCCCCHHHHHHHHHHHCCCCEEECCHHH
T.HGEEEETT..HHHHHHHHHHHHHHHHHHHHHHH..HHHHHHHHHHHHS..EE.HHHHH

--+-+----------++++++++-----+--------+++++++++++-------+++-+
--++++++-+------++-++----------------+-+-+------------------
-+++++++++------++++++++-----+-------+-+-++++-+-++++--+-+---
++++++++++------+++++++--------------++++++++++-++++--+-++--
eeee-eeeeeeeeeeeeee---eeeeeeeeeeeeeeee-ee--ee--eeeeee-eeee-e
eeee---eeeeeeee--e----e-eeee-eee-eeeee-e---ee---eeee--e-ee--
e--e---eeeeeeee--e----e-eeee-eee-e-eee-e---e------ee--e--e--
e--e---eeeeeeee--e----e-eeee-ee--e-eee-e---e------e---e-----
--------eeeeeee-----------ee-ee--e-e--------------e---------

1501 1560
DKLTSLAEADIIEELPEIIGRVTFPQAYQMINRDIGQLPLDIDDIKLIFRYCILNDPLMI
HHHHCHHHHHHHHHCCHHCCCCCHHHHHHHHHHHHCCCCCHHHHHHHHHHHEEECCCHEE
HHHHHHHHHHHHHH.HHHEEEE..HHHHHHHHHHTT.....HHHHHHHHHHHHHT.HHHH
++++++--++++--++---+-----++++++++----------+++++++++++-++-++
---------------------+----++---+-+----------++++++++++++++++
+------+-+---------+++++-++++-++-+---------+++++++++++++--+-
+++----+-+---------++++--++++----+---------+++++++++++++--++
ee-eeeeeee--ee-ee-eee-e-ee-eee-eee-ee-eee-ee-e---e----eee-e-
ee-ee--e-e--ee-ee---e-e-ee--e--eee-ee-e-e-ee-e--------e-e---
ee-ee--e-e--ee-ee---e-e-ee--e--eee-ee-e-e-ee-e----------e---
ee--e--e-e--ee-ee---e---e---e---e---e---e-e--e--------------
ee----------ee--e---------------e---e-----e-----------------

1561 1620
TAANTSLLCVKGTPQDRTGLSASQMPEFRNMKLIHHSPALVLKAFSKGTSDIPGADPIEL
EHHCCEEEEECCCCCCCCCCCCCCCHHHHHCHHECCCHHHHHHHHCCCCCCCCCCCHHHH
HHHH.EEEEET......H...GTT.HHHHHHHHH...HHHHHHHH.TT....TT..HHHH
+++-++++++-----------------++-++-----+++++++++----------+-+-
+--++++++++------------------+-+-----+--++++++---------++--+
+++++++++++------------------+++--++++--+++++---------------
+-+++++++++--------------------+----++--++++--------------+-
e-eeeee---eeeeeeeeeeeeeeeeeeee-eeeeeeeee--e-eeeeeeeeeeeeeee-
e-ee------eeeeeeeeeeeeee-ee-ee-e--ee-ee---e--eeeeee-eeeee-e-
---e------eeeeeeeee-eeee-ee-ee-e--ee--e---e--eeeeee-eeeee-e-
----------eeeeeeeee-e-ee-ee-e--e--e-------e--eeeeee-eeee--e-
----------e--eeeeee---e--ee-------e-------e--eeeeee-eee-----
◘

1621 1680
EKDLHHLNEFVETTAIKEKILHNIDNPPKHLIGNEILIYRIREMTKLYQVCYDYVKSTEH
HHHHHHHHHHHHHHHHHHHHHHHCCCCCCCCCCHHHHHHHHHHHHHHHHHHHHHHHCCCC
HHHHHHHHHHHHHHHHHHHHHH...S..G..HHHHHHHHHHHHHHHHHHHHHHHHH..ST
+-++-++++++----+++++++----------+-++++++++++++-+++++-++-----
+-+-------------+-++--+--------------+-+--------+-----------
+-+-------------+-+---------------+----+--------+-----------
+-+--------+----+-+--------------------+--------+-+---------
eeeeee-eeeeeeee-eee-eeeeeeeeeeeeeee---ee-ee-eeeee--eeeeeeeee
eee-ee-ee--e-e--eee--ee-eeeeee--eee----e-ee-ee--e---ee-eeeee
eee-ee-ee--e-e--eee--ee-eeeeee--eee------ee-ee--e---ee-eeeee
-ee-ee-ee--e-e--eee--ee-eee-ee-----------ee--e--e---e--eeeee
-e--e---e--------e---e--ee--e----------------e------e--e-eee

1681 1740
KVKIFILPMKSYTAIDFCTLIQGNTISDNKWYTMHYLKQIASGSIKGNIVTTSTSEQIIA
EEEEEEECCCCEEHHHHHHHHCCCCCCCCCHHHHHHHHHHHCCCCEEEEECCCHHHHHHH
EEEEEEEE..EEEHEEHHHHEET....STHHHHHHHHHHHHTT.ETE.EEE..HHHHHHH
-++++--------+-+++++++--------++++++++++++-+--+++-------++++
++++-----------++-+--+-+-------+++++++++-----+-+++-----++--+
+-++-----------++----+---------++++++++++----++--------++--+

```
++++-----------+--+-------------+---+++------+---------++--+
e-e---eeeeeee--e--e--eeee-eeeeee--ee-ee--eeeeeeeeeeeeeeeeeee
e-e----e-ee-e--------eeee-eeee----e--ee--eee-eee-eeeeeeee---
e-e----e-ee----------eeee-eeee----e--ee--eee-eee--eeeeeee---
e-e------e-----------e--e--eee----e--ee--eee-e-e--eeeeeee---
e--------e------------------e------------e---e-----eee-ee---
◘

1741                                                    1800
NECFRVLCHFADSFVEEASRLSFINEVLDNFTYKNISVNSLFNTLLASTTRLDFIPLLFR
HHHHHHHHHHHHHHHHHHHHHHHHHHHHHHCCCCCCEHHHHHHHHHHCCCCCCHHHHHHH
HHHHHHHHHHHHHHHHHHHHHHHHHHHHHH.EEEEEEHHHHHHHHHHHH.HH.HHHHHHH
+++++++++++++++-+++++++++++++----------+++++++-------++++++-
-+-++++++-++------------------------------+-------+-+--+--++
++++++++++++-----++++---------------------+-+-----+-++-+++++
+++++++++++++---++++++-++++---------+---+++-+-----+-+--+++++
ee--e---ee-eeeeee-eeeee-ee-eee-eeee-e-ee--ee-eeeeeeee--e---e
ee--e---e--ee-eee-ee-e--ee--ee-e-ee-e-ee--e---ee-ee-e------e
ee--e---e--ee--ee-ee-e--ee--ee-e-ee---e---e---ee-ee-e------e
ee--e------ee--ee-ee----ee--ee-e-e----e-------e--ee---------
-----------e---e---e-----e--ee---e--------------------------

1801                                                    1860
LKVLTQTDLNRFDALKTNERVSWNNWQTNRSLNSGLIDLTISGYLRSIRVVGEDNKLKIA
HHHCCHCCHHHHHHCCCCCCEECCCCCCCCCCCCCCEEEEECCCCEEEEEECCCCCCEEE
HHHHHH..HHHHHHHHH..EEEET...H.....TT.EEEEEEEEEEEEEEE.TTTEEEEE
+------++-+++++-------------------++++++++++++++-+-+-----+++
--------------------------+-----++--+++++++++++++++++-++++++
+---------+-----------------++--+--++++++++++++++++++--+++++
++-------------------+----------+--++++++++++++++++++--+-+-+
-ee-eeeeeeeeeeeeeeeeeeeeeeeeeeeeeeee-e-e-eee-e--e-eeeeeeee--
-e--eeeeeee-ee-eeeee-e-eeeeeeee-eee--e-e-e-e-e--e--eeeee-e--
-e--eeee-ee-ee-eeeee---ee-eeeee-ee---e-------e--e---eeee-e--
-e--eeee-ee-ee-eeeee---ee-eeeee-ee---e--------------eeee-e--
------e--e--e--eeeee---e---e-e----------------------ee-e----
◘

1861                                                    1920
ELTIPNFYPNTVFHAGNKLLNSRHGLKFEYMEEIVLDEKYNYYITYQKKRAHIYTYQVST
EEECCCCCCCCEEECCHHHHCCCCCCCECCHHEEEECCCEEEEEEEECCCCEEEEEEECC
EEE..T..TTHEEEETHHHHH.TTT.EHHHHHHEEEHTTEEEEEEEEEEEEEEEEEEHHH
+---------+++-++++++++--------+-++--+--+++++++------+-+++---
+++-------++++++++-+------+---+---------+--++--------++++++-
+++-------++++++++--------+-+-+++-------++++++++---+--+++++-
+++--------++-+++---------+-+-+++-------+++++--------+++++++
e-e-eeeeee---eeeeee-eeeee-e-ee-ee-eeeeeeeee-eeeeeeee-eeeeeee
e-e-ee-eee---e--ee--eeeee-e-e--ee---eee-e---e-eeee-e----e-ee
e-e-ee--e----e--ee--eeeee-e-e--ee---eee-e-----eeee------e---
e-e-ee--e--------e--eeee--e-e--ee---eee-------eeee----------
-----------------e----e--------------ee--------ee-----------

              L4

1921                                                    1980
IEHILRRNNEGLQSRGPRYNKMVPVCPVVLSVRDELFRMSLENVFSLNMTNFSMSRLFVS
HHHHHHHHCCCCCCCCCCCCCCCCCCEEEEEEECCCCEEEEHCHHHCCCCCCCCEEEEEC
HHHHHHHH.TT.........T......EEEEEHHHHHHHHHHHHHHHHHHH..HHHHE..
-+++++++------------------+---+--+--++++++++++-+----+++++---
-------+-----------+---++-++++++-----+-+------------------+-
-+--+++----------------++-+++++------+-+-------------+-++---
-+--+------------------+-+++++++-----+-++++---------+-++++--
```

```
-ee--eeeeeeeeeeeeeeeeeeee-e---e-eee--eee-ee--e-eeeeee-ee-eee
-ee--eeeeee-eeeeeeeeee-ee-e---e-eee--e---ee--e-e-ee-e--e----
-e---eeeeee-eeeeee-eee-e--e-----eee--e---ee----e-ee----e----
-e---eeeeee-eeeeee-eee-e---------ee--e---e-----e-ee----e----
-----e--ee--eee-ee-ee--------------------e------------------
```

```
1981                                                    2040
PDEVATVKKAHMSKMMFFSGPTIKAGIINLTSLMRTQELLTLNYDNLCKSSIVPFCRILE
CCHHHEHHHHHHHEEECCCCCCCECEEEHHHHHHHHHHHHHCCCHHCCCCCCCCHHEEEE
THHHHHHHHHHHHHHHHHHSHHHHHEEEEHHHHHHHHHHHHHHHHTT....HHHHHHHEE
--++++++++++++++--------+++++++++++++++++-++-+-------++++++-
-----+++-----+-+------++-+--+----+--+-----+----------+++++++
-------++-+--+---------+-+--++----+-------+---+-+----+++++++
--+--+-++----+---------+-++-++----+-++----+-------+--+++++++
eeeeee-eeee-ee---eeeee-ee---e-eeeeeeeee-eeeeeee-eeeeee--e--e
eee--e-eeee-ee----eeee-e----e--e-ee-ee--e-eeeee-eeee-e--e--e
eee--e-ee-e-ee----eeee-e-------e--e-ee--e-e-ee--eee--e--e--e
eee--e-ee----e------e--e----------e-ee--e-e-ee--e-------e--e
ee------e----e---------e--------------------ee--e----------e
```

```
     L5

2041                                                    2100
CNGDEQGELIFLSDEVMDFTISEEIESMPLFTIRYQKRGTEIMTYKNAIMKLVSAGVDEI
ECCCCCCEEEECCCCCCCCCCCHHCCCCEEEEEEEECCCCEHHHHHHHHHHHHHHCCCHE
ETT.TT.EEEEE.HHH.EEEE.HHHHTS.EEEEEEE.TT.HHHHHHHHHHHHHHTT.HHH
----------+-+-----------+-----++++---------++++++++++---+-++
++-----++++++------------------+++++------+-+++++--++-------
--------+++++--------------------++---------++++++-+--------
-+------+++++------------------++++----------+++++++--------
eeeeeeee-----ee-eeee-eee-eeeee-e-eeeeeeeeeee-ee--ee--eeeeee-
-eeeeeee-----ee--e-e-eee-ee-ee---e-eeeeee--e-ee---e--eee-ee-
-eeeeeee-----ee--e-e-eee-ee-e----e-eeeeee--e-ee---e--eee-ee-
-eeeee-e-----ee--e-e--ee-ee------e--eeeee----e----e--eee-ee-
-eeeee--------e-------e--ee---------ee------------e---e--ee-
◘
```

```
2101                                                    2160
KEVFDFSKQGFYSKKNLGIINTICSIINILETNEWSTILYNSFHIAMLLESMDREFHMFT
HEHECCCCCCCCCHHHHHHHHHHHHHHHHHHCCHHHHHHHHHHHHHHHHHCCCCCCCCCC
HEHEEHHHHT.E.HHHHHHHHHHHHHHHHHHH.HHHHHHHHHHHHHHHHHHHHHHHHHE.
+++++-+---------++++++++++++++-----++++++++++++++--++-------
+-+------------++++++++++--+-++----+++--++++++++--------+---
+--------------+++++++++++-+-++----++--++++++++++-------+---
+---------------++--++++++++--+----++-+++++++++++-----------
eeeeeeeeeeeeeeeeee--e------e-eeeeee-e--e---------ee-eee-e--e
ee--e-eeee-eeeee----e------e--eeee---------------e--eee----e
ee--e-eeee-e-eee---------------eee---------------e--eee-----
ee--e-eee----ee-----------------ee---------------e--eee-----
ee--e--ee----ee---------------------------------------------
```

```
2161                                                    2220
LPEAFFINVAGGVVNWTKLLKFIKSLPVIEQEPWSMMMSRFVEKTVYLIEREMNKDVDFT
CCCEEEECCCCCCHHHHHHHHHHHCCCCCCCCCCHHHHHHHHHHHHHHHHHHHCCCCCHH
..HHHHEEHHTHHHHHHHHHHHHHTS..EEE..HHHHHHHHHHHHHHHHHHHHHT...HH
-+-+++-++-----++++++++++---------+-+++-++++++++++++-+-------
-+++-++++-----++-+-------------------------+++-+++----------
```

```
--+++++++-----+--++----------------------+++++-+++----------
-+++-++++-----+++++++-+-------------------++++-+++----------
-ee----e-ee---e-ee--ee-eeeeeeeeeeeeee-eee-ee--e--eeeeeeeee-e
-ee-----------e--e--e--ee-ee-eeee-ee---e--ee--e--eee-eee-e-e
-----------------e--e--ee-ee-eeee-e----e--ee------ee-eee-e-e
-----------------e--e--ee-e--ee-e------e--ee------ee-eee-e--
-----------------e--e--e--e--ee-------------------e--eee-e--



2221           2238
DFLDELEFSSGKSLFTFF
HHHHHHHCCCCCCEEEC
HHHHHHEE.TS.EEEEE.
+++++++-----------
------+-----------
------------------
------------------
eeeee-eeeeeeee-eee
e--ee-e-eeeee--eee
e--ee-e-eeeee--e-e
e--ee-e-eeee---e-e
---e-----eee---e-e
□□
```

The 8 lines have the following meaning:

* Line 1: The 1-letter code of your protein primary sequence. This line is always present.
* Line 2: Secondary structure prediction:

H = helix
E = strand
C = the rest
This line is present if you requested an SSpro prediction.

* Line 3: 8-class secondary structure prediction:

H: alpha-helix
G: 310-helix
I: pi-helix (extremely rare)
E: extended strand
B: beta-bridge
T: turn
S: bend
C: the rest
This line is present if you requested an SSpro8 prediction.

* Lines 4-7: Predictions of number of residue contacts at 6, 8, 10 and 12 Å:

\+ : the residue has more contacts than its average
\- : the residue has fewer contacts than its average

· The 4 lines are present if you requested a CONpro prediction.
· Line 8: Prediction of relative solvent accessibility:
· - : the residue is buried
· e : the residue is exposed

The threshold of solvent accessibility used to discriminate the buried/exposed state is reported right after the prediction.

This line (or lines, in case multiple thresholds are selected) is present if you requested an ACCpro prediction and the buried/exposed threshold is the one you selected, or the default 10%, 20%, 25%, 30% and 40%.

SSpro, ACCpro, CONpro, SSpro8, CMapPro and CMap23Dpro can be found at:
http://www.igb.uci.edu/tools/scratch/

For an explanation of the output formats, refer to:
http://www.igb.uci.edu/tools/scratch/explanation.html#output_formats

For the SCRATCH web server and a general overview please cite:

J. Cheng, A. Randall, M. Sweredoski, P. Baldi, SCRATCH: a Protein Structure and Structural Feature Prediction Server, Nucleic Acids Research, vol. 33 (web server issue), w72-76, 2005.

P. Baldi and G. Pollastri, "The Principled Design of Large-Scale Recursive Neural Network Architectures-DAG-RNNs and the Protein Structure Prediction Problem", Journal of Machine Learning Research, 4, 575-603, (2003).

Supplementary Fig. S1. (a) Alignment of the L proteins of bunyamwera, oropouche and La Crosse orthobunyaviruses. (b) Bioinformatic analysis of Bunyamwera virus L protein.

Supplementary Table S1. Densitometric analysis of the BUNV N and L protein bands in Fig. 6(b)

The amounts of protein in each band were estimated by scanning the gel in a phosphorimager (FLA-5000; Fujifilm Corporation) and use of density analysis software (Image Gauge, version 4.21; Fujifilm Corporation and Koshin Graphic Systems, Inc.). The degree of co-immunoprecipitation was assessed by normalizing the density of N protein band (lanes 1–4) or L protein band (lanes 5, 6), and then correcting the density in the co-precipitated band.

Lane no.	Density of N band (arbitrary units)	Density of L band (arbitrary units)	Density of L band after normalization of N band	Density of N after normalization of L band
1	18495	11	11	
2	4313	22	94	
3	18361	35	35	
4	1248	9	133	
5	20	1096		26
6	118	1151		138

Shi, X. and Elliott, R. M. (2009). Generation and analysis of recombinant bunyamwera orthobunyaviruses expressing V5 epitope-tagged L proteins. *J Gen Virol* **90**, 297–306.
